# Multiple factors and features dictate the selective production of ct-siRNA in *Arabidopsis*

**DOI:** 10.1038/s42003-024-06142-4

**Published:** 2024-04-18

**Authors:** Li Feng, Wei Yan, Xianli Tang, Huihui Wu, Yajie Pan, Dongdong Lu, Qianyan Ling-hu, Yuelin Liu, Yongqi Liu, Xiehai Song, Muhammad Ali, Liang Fang, Hongwei Guo, Bosheng Li

**Affiliations:** 1 Peking University Institute of Advanced Agricultural Sciences, Shandong Laboratory of Advanced Agriculture Sciences in Weifang, Weifang, Shandong 261325 China; 2 Institute of Plant and Food Science, Department of Biology, Southern University of Science and Technology, Shenzhen, Guangdong 518055 China

**Keywords:** RNA decay, siRNAs

## Abstract

Coding transcript-derived siRNAs (ct-siRNAs) produced from specific endogenous loci can suppress the translation of their source genes to balance plant growth and stress response. In this study, we generated *Arabidopsis* mutants with deficiencies in RNA decay and/or post-transcriptional gene silencing (PTGS) pathways and performed comparative sRNA-seq analysis, revealing that multiple RNA decay and PTGS factors impede the ct-siRNA selective production. Genes that produce ct-siRNAs often show increased or unchanged expression and typically have higher GC content in sequence composition. The growth and development of plants can perturb the dynamic accumulation of ct-siRNAs from different gene loci. Two nitrate reductase genes, *NIA1* and *NIA2*, produce massive amounts of 22-nt ct-siRNAs and are highly expressed in a subtype of mesophyll cells where *DCL2* exhibits higher expression relative to *DCL4*, suggesting a potential role of cell-specific expression of ct-siRNAs. Overall, our findings unveil the multifaceted factors and features involved in the selective production and regulation of ct-siRNAs and enrich our understanding of gene silencing process in plants.

## Abstract (web summary)

Research on *Arabidopsis* indicates that RNA decay, PTGS pathway, GC content, and spatial-temporal gene expression impact ct-siRNA production, vital for gene silencing during plant growth and stress responses.

## Introduction

In various eukaryotes, small non-coding RNAs (sRNAs) play crucial roles in silencing genes at both post-transcriptional (PTGS) and transcriptional (TGS) levels^1,2^. Plant sRNA population is mainly classified into two groups: microRNAs (miRNAs) and small interfering RNAs (siRNAs). Among these, miRNAs generate from precursors transcribed from *MIRNA* genes by RNA polymerase II (Pol II) and cleaved by DCL1. In contrast, siRNAs are primarily derived from double-stranded RNAs (dsRNAs) that are catalyzed by RNA-dependent RNA Polymerase (RDR) proteins from the transcripts of transposons, transgenes, and viral RNAs^3^. Generally, DCL4 is responsible for cleaving most RDR6-dependent dsRNAs into 21-nt secondary siRNAs. Subsequently, the mature miRNAs and siRNAs are loaded into Argonaute (AGO) proteins to form RNA-induced silencing complexes (RISC), which can mediate the cleavage or translational repression of target genes^4^.

A novel class of siRNAs known as coding transcript-derived siRNAs (ct-siRNAs) has been discovered in *Arabidopsis* plants deficient in various RNA decay factors^5^. RNA decay is a complex process that involves the 5’-3’ exonuclease XRN (EXORIBONUCLEASE) and the 3’-5’ multi-subunit exonuclease protein complex (exosome)^6,7^. The 5’-3’ or 3’-5’ RNA decay can occur in both the nucleus and cytoplasm, with different cofactors involved. In *Arabidopsis*, the cytoplasmic XRN4/EIN5 mainly degrades uncapped mRNAs and RNA fragments cleaved by miRISC/siRISC^8^, while XRN2 and XRN3 in the nucleus remove abnormal RNAs during transcription^9–11^. Loss of EIN5, SKI2, and XRN3 proteins can lead to the production of massive ct-siRNAs^11–13^. As exosome cofactors, members of Superkiller complex (SKI complex), such as SKI2, SKI3, SKI7, and SKI8, are required for 3’-5’ cytoplasmic RNA decay^7^. The homologous nuclear-localized MTR4 assists in removing rRNA precursors and maturation by-products, while the nucleocytoplasmic-localized HEN2 is involved in the degradation of polyadenylated nuclear exosome substrates^14–16^. A recent study detected distinct accumulation patterns of ct-siRNAs at miRNA targets in plants deficient in the HEN2 and SKI2 activity^17^. Other exosome cofactors with unknown functions have also been identified in *Arabidopsis*, such as RST1 and RIPR, and deficiency of these factors can lead to the production of ct-siRNAs from over one hundred endogenous coding transcripts^18,19^.

RNA decay is a multi-step process that involves removing the poly(A) tail, mediated by the CCR4-NOT complex and PAN2-PAN3, followed by the binding of the shortened poly(A) by LSM1-7(SM-like)/PAT1 and the recruitment of the mRNA decapping complex. After the removal of 5’ cap, mRNAs can be directionally degraded either from the 5’-3’ by EIN5 or 3’-5’ by exosome^20,21^. The poly(A) truncation process is often accompanied by modifications to the poly(A) tail. In plants, specific mRNAs undergoing poly(A) truncation are initially uridylated by Uridylyltransferase 1 (URT1) before being degraded in the directional 5’-3’ manner, thereby inhibits 3’-5’ RNA decay^22^.

The decapping complex, which is responsible for removing the canonical 5’ m^7^G cap of mRNAs, consists of Nudix family members and Decapping 2 (DCP2) cofactors, such as DCP1 and DHH1^23^. DCP1, DCP2, DCP5, and VARICOSE (VCS) have been found to play important roles in post-embryonic plants^24,25^. Additionally, mRNAs carrying non-canonical 5’ NAD^+^ caps can be degraded by the non-Nudix family hydrolase Decapping and exoribonuclease protein 1 (DXO1)^26–28^. Plants deficient in URT1, DCP2, VCS, and DXO1 enhanced the accumulation of ct-siRNAs^27,29,30^. FRY1 (FIERY1) is another 5’-3’ RNA decay factor that promotes the abundance and function of miRNAs by inhibiting the biogenesis of ribosomal RNA-derived siRNAs (risiRNAs)^12^. Therefore, RNA decay factors intricately coordinate to regulate the fate of mRNAs, ensuring normal gene expression by preventing aberrant mRNAs from being captured by the PTGS pathway.

Our previous study found that in *ein5 ski2* plants, a minimum of 441 protein-coding genes can produce ct-siRNAs, mainly 21-nt in length. The biogenesis of these ct-siRNAs relied on DCL4/DCL2, RDR6, and SGS3, with partial dependence on AGO1^13^. In contrast, in *ein5 dcl4* and *ski2 dcl4* plants, a massive amount of RDR6- and DCL2-dependent 22-nt ct-siRNAs accumulated, leading to more severe growth and developmental defects^31^. Approximately 50% of the total 22-nt ct-siRNAs originated from *NIA1* and *NIA2*. These ct-siRNAs are predominantly loaded into AGO1, leading to the inhibition of NIA1 and NIA2 protein levels by stimulating secondary siRNA amplification and inducing strong gene silencing effects^31^. Highly abundant 22-nt ct-siRNAs play a crucial role in regulating plant responses to nitrogen deficiency, ABA signaling, and salt stress^31^. Although the source genes of ct-siRNAs represent only a small portion of the genome-wide expressed genes, the distinct accumulation of 22-nt ct-siRNAs at different loci in plants deficient in RNA decay and PTGS factors or under various stresses suggests that ct-siRNA production is regulated by unknown selective and regulatory mechanisms. A previous study reported that the 5’-3’ RNA decay factor EIN5 selectively degrades *cis*-acting elements containing the CTCCGT motif, thereby more effectively preventing them as substrates for ct-siRNA production^32^. Additionally, transgenes characterized by a high GC content in their sequence composition have been observed to enhance protein translation rates and slowdown RNA degradation in plants by modulating the codon-tRNA matching efficiency^32^. Therefore, studying the characteristics of source genes is essential for understanding the determining mechanisms of ct-siRNA selective production from distinct endogenous coding genes in plants.

In this study, our aim was to elucidate the selective production and regulatory mechanism of ct-siRNAs. To achieve this, we constructed a series of mutants with deficiencies in RNA decay and PTGS factors, followed by performing sRNA-seq, RNA-seq, and single-nucleus RNA-seq (snRNA-seq). Comparative analysis revealed that multiple RNA decay and PTGS factors strongly inhibit the ct-siRNA selective production. Genes with high GC content in their sequence composition contribute to the accumulation of highly abundant ct-siRNAs. Transgenic experiments involving truncated *NIA1* and *NIA2* fragments suggested that ct-siRNA-induced off-target silencing may lead to the transitive silencing of *NIA1* and *NIA2*. Additionally, we unveiled the importance of the spatiotemporal expression of ct-siRNA source genes at both the developmental stage and single-cell level in the selective production of ct-siRNAs. Overall, our study advances our understanding of RNA silencing and provides new insights into the role of ct-siRNAs in regulating plant development and responses to stress.

## Results

### RNA decay and PTGS factors regulate 21-nt and 22-nt ct-siRNA selective production

**Fig. 1:**
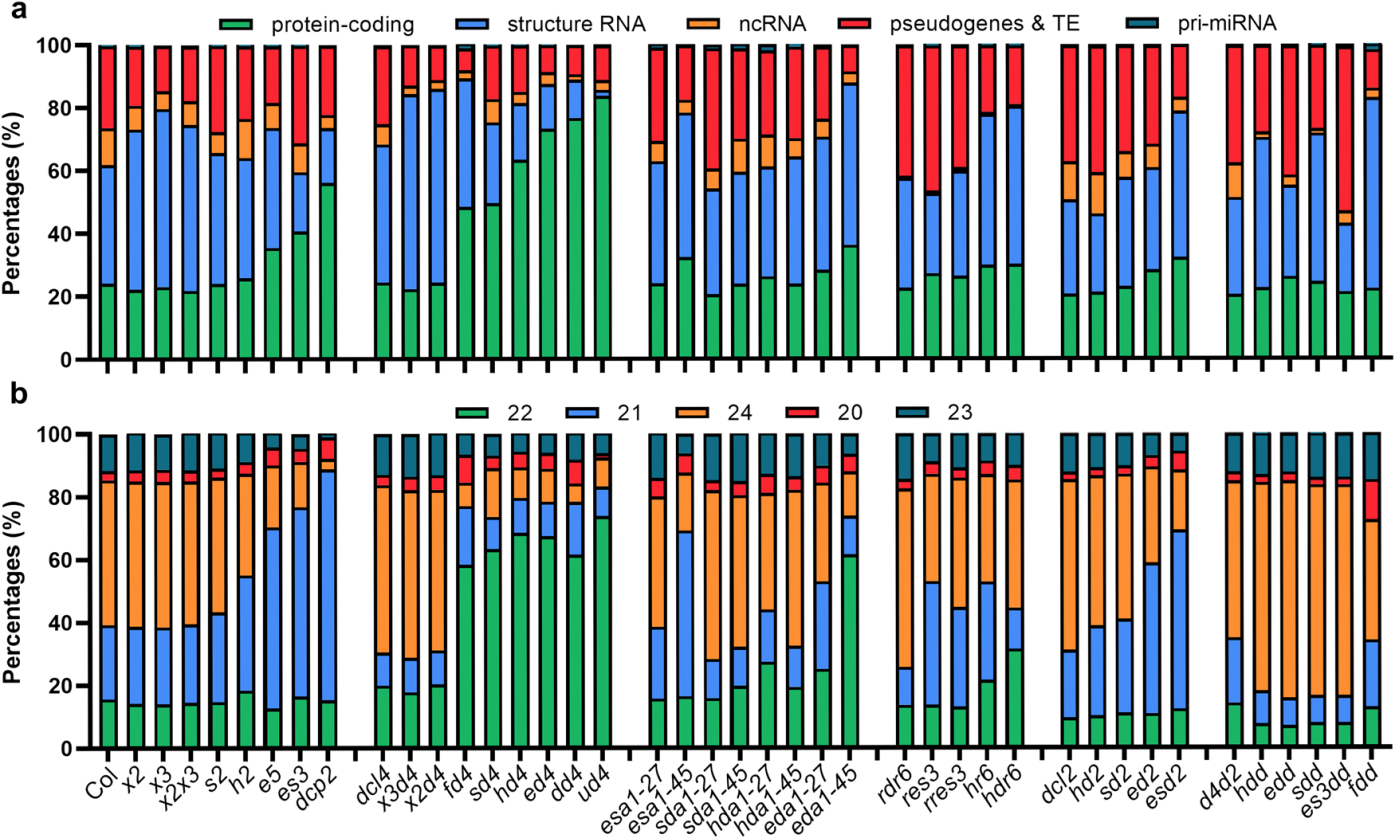
RNA decay and PTGS factors regulate ct-siRNA production. **a** The percentage of siRNAs derived from various siRNA-generating loci, including protein-coding, structure RNA, non-coding RNA, pseudogenes & TE, and pri-miRNA. The mutant alleles used in this study are abbreviated as *x2* (*xrn2-2*), *x3* (*xrn3-3*), *x2×3*(*xrn2-2 xrn3-3*), *s2* (*ski2-2*), *h2* (*hen2-1*), *e5* (*ein5-1*), *es3* (*ein5-1 ski2-3*), *dcp2* (*dcp2-1*), *dcl4* (*dcl4-2*), *x3d4* (*xrn3-3 dcl4-2*), *x2d4* (*xrn2-2 dcl4-2*), *fd4* (*fry1-6 dcl4-2*), *sd4* (*ski2-2 dcl4-2*), *hd4* (*hen2-1 dcl4-2*), *ed4* (*ein5-1 dcl4-2*), *dd4* (*dxo1-2 dcl4-2*), *ud4* (*urt1-1 dcl4-2*), *esa1-27* (*ein5-1 ski2-3 ago1-27*), *esa1-45* (*ein5-1 ski2-3 ago1-45*), *sda1-27* (*ski2-2 dcl4-2 ago1-27*), *sda-45* (*ski2-2 dcl4-2 ago1-45*), *hda1-27* (*hen2-1 dcl4-2 ago1-27*), *hda1-45* (*hen2-1 dcl4-2 ago1-45*), *eda1-27* (*ein5-1 dcl4-2 ago1-27*), *eda-45* (*ein5-1 dcl4-2 ago1-45*), *rdr6* (*rdr6-11*), *res3* (*ein5-1 ski2-3 rdr6-11*), *rres3* (*ein5-1 ski2-3 rdr1-1 rdr6-11*), *hr6* (*hen2-1 rdr6-11*), *hdr6* (*hen2-1 dcl4-2 rdr6-11*), *dcl2* (*dcl2-1*), *hd2* (*hen2-1 dcl2-1*), *sd2* (*ski2-2 dcl2-1*), *ed2* (*ein5-1 dcl2-1*), *esd2* (*ein5-1 ski2-3 dcl2-1*), *d4d2* (*dcl4-2 dcl2-1*), *hdd* (*hen2-1 dcl4-2 dcl2-1*), *edd* (*ein5-1 dcl4-2 dcl2-1*), *sdd* (*ski2-2 dcl4-2 dcl2-1*), *es3dd* (*ein5-1 ski2-3 dcl4-2 dcl2-1*), and *fdd* (*fry1-6 dcl2-1 dcl4-2*). **b** The percentage of ct-siRNAs with lengths ranging from 20-nt to 24-nt. Only reads produced from the antisense strand of protein-coding genes, representing ct-siRNAs, were calculated.

Our previous study demonstrated that loss of the cytoplasmic RNA decay and/or DCL4 activity in *Arabidopsis* induces the production of abundant DCL2-dependent 22-nt ct-siRNAs from specific endogenous loci, leading to the silencing of their source genes and the defects of plant growth and development^31^. To investigate the selective production and regulatory mechanism of ct-siRNAs, we generated a series of mutants with deficiencies in RNA decay and/or PTGS factors and performed sRNA sequencing. By analyzing the abundance of siRNAs accumulated in these mutants, we found that RNA decay and PTGS factors can markedly suppress the production of siRNAs from protein-coding loci (Fig. 1a, Supplementary Fig. 1a). Mutation of *FRY1* could induce the biogenesis of risiRNAs from ribosomal RNAs, which belong to a class of structural RNAs^12^. Notably, there is a reciprocal relationship between the expression levels of siRNAs produced from structural RNAs and coding genes in these mutants. Minor changes in the accumulation of siRNAs from other genomic regions suggest that endogenous mRNAs might preferentially entering the PTGS pathway as substitute substrates for structural RNAs (Fig. 1a, Supplementary Fig. 1a).

Given that sRNAs generated from the sense strand of their source genes can represent either functional siRNAs or RNA degradation fragments, while those originating from the antisense strand are typically considered as genuine siRNAs processed by RDR6 and DCLs, we particularly focused on antisense strand-derived ct-siRNAs. We observed that mutants with defects in RNA decay and/or PTGS pathways produced varying levels of ct-siRNAs, mainly 21-nt and 22-nt in length (Fig. 1b, Supplementary Fig. 1b). These ct-siRNAs significantly accumulated in eight mutants, including *ein5-1 ski2-3*, *dcp2*, *fry1-6 dcl4-2*, *ski2-2 dcl4-2*, *hen2-1 dcl4-2*, *ein5-1 dcl4-2*, *dxo1-1 dcl4-2*, and *urt1-1 dcl4-2*. Among these mutants, *dcp2* exhibited the most pronounced accumulation of 21-nt ct-siRNAs, followed by *ein5-1 ski2-3*, while the other six mutants with DCL4 defects were mainly enriched in 22-nt ct-siRNAs (Fig. 1b, Supplementary Fig. 1b). We also found that 21-nt ct-siRNA biogenesis relied on DCL4, AGO1, and RDR6, and could shift to 22-nt or 24-nt when DCL4 was deficient, or when both DCL2 and DCL4 functions were simultaneously lost (Fig. 1b, Supplementary Fig. 1b). These results suggested that DCL proteins, including DCL4 and DCL2, competitively inhibit ct-siRNA production in plants deficient in RNA decay. Indeed, DCL2 is typically considered less competitive than DCL4 in processing RDR6-dependent dsRNAs into siRNAs. However, compared to the *ein5-1 ski2-3* mutant, the abundance of 21-nt ct-siRNAs declined in the *ein5-1 ski2-3 dcl2-1* plants although it remained at higher levels than in the wild-type Col-0 and *ein5-1 ski2-3 dcl4-2 dcl2-1* mutant (Supplementary Fig. 1b). This suggests that DCL2 could still contribute to the production of 21-nt ct-siRNAs even when DCL4 is functional. Therefore, our findings demonstrate that RNA decay and PTGS factors impede the selective biogenesis of 21-nt and 22-nt ct-siRNAs to varying levels and exhibit cumulative effects.

### Dispersion of ct-siRNAs in mutants deficient in RNA decay and PTGS pathways

**Fig. 2:**
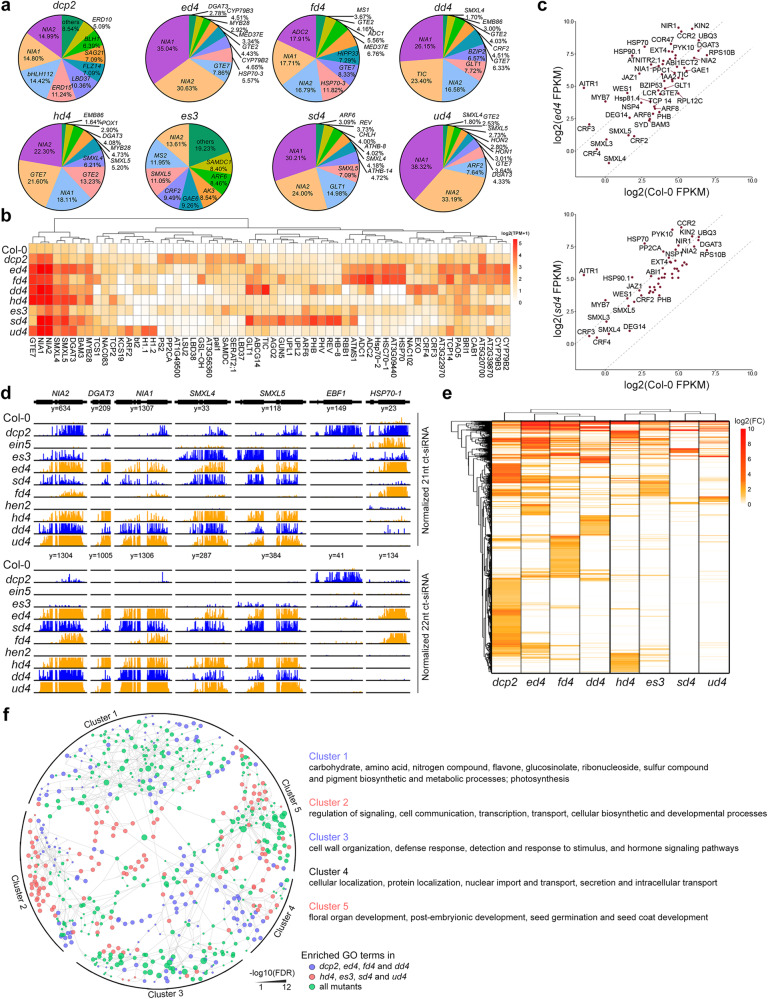
Accumulation and distribution of ct-siRNAs in mutants deficient in RNA decay and PTGS factors. **a** Pie charts ranking the top10-scoring 22-nt ct-siRNA-producing loci by accumulated ct-siRNA abundance. **b** Heatmap depicting the expression of ct-siRNAs produced by 52 hotspot genes. **c** Dot plot of the relative expression levels (log2) of hotspot genes in *ed4* (*ein5 dcl4-2*) or *sd4* (*ski2-2 dcl4-2*) mutant versus Col-0 plants. **d** An Integrated Genome View (IGV) illustrating the distribution of 21-nt and 22-nt ct-siRNAs accumulated at specific genes. **e** Clustering of samples based on the relative expression of ct-siRNAs in mutants versus Col-0. **f** A Gene Ontology (GO) annotation network illustrating the function categories of ct-siRNA source genes influenced by different groups of RNA decay factors.

Our recent study revealed that 22-nt ct-siRNAs could strongly inhibit the translation of their source genes instead of cleaving the transcripts^31^. In this study, we tried to identify genome-wide hotspot genes that repeatedly producing high levels of 22-nt ct-siRNAs among the eight mutants mentioned above. In double mutants deficient in RNA decay and DCL4 activity, including *ein5 dcl4*, *dxo1 dcl4*, *hen2 dcl4*, *ski2 dcl4*, and *urt1 dcl4*, *NIA1* and *NIA2* produced almost half of the 22-nt ct-siRNAs among the top 20 ct-siRNA source genes (Fig. 2a). Other loci, such as *DIACYLGLYCEROL ACYLTRANSFERASE 3* (*DGAT3*), *GLOBAL TRANSCRIPTION FACTOR GROUP E 2/7* (*GTE2/7*), and *SMAX1-LIKE 4/5* (*SMXL4/5*), consistently contributed a high percentage of 22-nt ct-siRNAs (Fig. 2a).

Considering the amounts of 22-nt ct-siRNAs derived from the top 20 source genes account for more than 80% of the total 22-nt ct-siRNAs (Fig. 2a), all the top 20 source genes in the eight mutants were regarded as hotspot genes producing 22-nt ct-siRNAs, resulting in a union set of 52 genes (Fig. 2b). Analyzing the expression patterns, functions, and sequence features of these hotspot genes will assist in elucidating the potential mechanism of ct-siRNA selective production. Consistent with our previous findings, mRNA levels of these hotspot genes remained unchanged or were even up-regulated in *ein5-1 dcl4-2* and *ski2-2 dcl4-2* plants (Fig. 2c), leading us to further estimate the dynamic production of ct-siRNAs during plant growth and development, as well as the expression patterns of source genes in different cell types later in this work.

While there was considerable overlap in the genes producing 22-nt ct-siRNAs among the eight mutants, we still found several loci specifically generating this class of siRNAs in less than three mutants (Fig. 2d). For example, 21-nt and 22-nt ct-siRNAs originating from *EBF1* were only detected in *dcp2* and *ein5-1 ski2-3*, while 22-nt ct-siRNAs generating from *HSP70-1* were specifically detected in *ein5-1 dcl4-2* and *fry1-6 dcl4-2* (Fig. 2d). The abundance of 21-nt ct-siRNAs were also estimated for the selected genes for comparisons with 22-nt ct-siRNAs (Fig. 2d). The distinct accumulations of 21-nt and 22-nt ct-siRNAs at several loci in different mutants indicated diverse inhibitory effects of specific RNA decay and PTGS factors on ct-siRNA production. To validate this hypothesis, we assessed the genome-wide production of 22-nt ct-siRNAs in all the eight mutants. Compared with Col-0, the profiles of 22-nt ct-siRNAs displayed substantial variations among the eight mutants, with those deficient in 5’-3’ and 3’-5’ mRNA decay being clustered into two distinct clusters (Fig. 2e). Despite the shared biological functions among the ct-siRNA source genes, the genes in mutants deficient in 5’-3’ mRNA decay were mainly enriched in photosynthesis, metabolic processes, and defense-related functions, while genes in mutants affecting 3’-5’ mRNA decay were specifically enriched in signaling regulation, cell communication and organ development-related functions (Fig. 2f). These findings suggest that RNA decay and PTGS factors can specifically or synergistically inhibit the selective production of ct-siRNAs. The dispersed accumulation of ct-siRNAs at specific genes suggests that RNA decay and PTGS factors regulate ct-siRNA selective production at both quantity and functional levels.

### Source gene characteristics contributing to ct-siRNA selective production

To investigate the potential features of ct-siRNA source genes, we focused on their biological functions, expression levels, and sequence characteristics in the eight mutants mentioned above. In double mutants deficient in RNA decay and/or DCL4 activity, the production of ct-siRNAs was increased to varying levels among source genes. We analyzed the biological functions of genes with similar fold-change ranges in ct-siRNA accumulation (Supplementary Fig. 2). Interestingly, the infrequent overlap among Gene Ontology (GO) terms annotated by genes exhibiting different fold-change scales indicates that the selective production of ct-siRNAs is related to the functions of their source genes (Supplementary Fig. 2). Notably, genes with a thousand-fold increase in ct-siRNA production (log2FC range 11-14.5 in Supplementary Fig. 2) were involved in processes such as nitric oxide biosynthesis, nitrate assimilation, or stress response to light or hormone stimuli, whereas genes with slightly increased ct-siRNA accumulations (log2FC range 2-5) tended to regulate cell death, photosynthesis, auxin and hormone transport, and development (Supplementary Fig. 2). In contrast, when plants deficient in RNA decay and DCL4 activity, no significant difference was found in the expression levels of genes producing 22-nt ct-siRNAs compared to those not producing them (Supplementary Fig. 3a). These findings suggest that the accumulation of ct-siRNAs correlates with the biological functions of their source genes rather than expression levels.

**Fig. 3:**
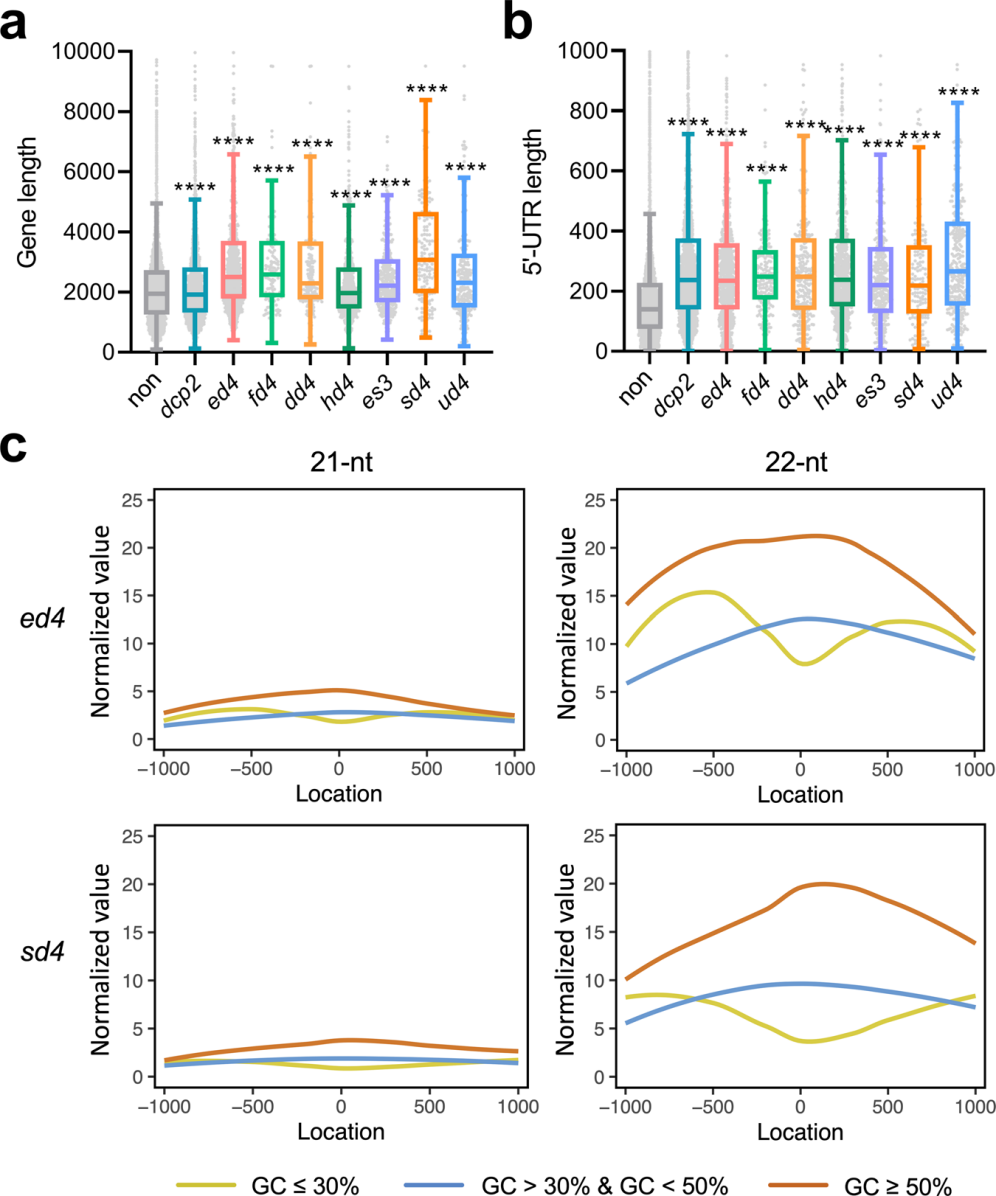
Impact of source gene characteristics on ct-siRNA production. **a** The sequence length distribution of source genes. **b** The 5’ UTR length distribution of source genes. **c** The abundance of ct-siRNAs derived from genes with low, medium, and high GC content, as well as their 1Kb upstream and downstream regions. A total of 3838 genes exhibited differential accumulation of 22-nt ct-siRNAs in the eight selected mutants when compared to Col-0, with a *padj* < 0.05 and log2FC (Fold Change) > 1. The curves figures in the left and right represent 21-nt and 22-nt ct-siRNAs, respectively.

Regarding the ct-siRNA generation in relation to the sequence composition of the source gene, we found that compared to genes unable to produce 22-nt ct-siRNAs, genes producing them tended to have longer sequences and extended 5’ UTRs (Fig. 3a, b), while no significant difference was observed in the 3’ UTR length and intron number (Supplementary Fig. 3b, c). Higher GC content in bacteria-originated transgenes can enhance expression and protein accumulation through mechanisms such as decreased mRNA degradation, improved translation efficiency, and optimized epigenetic modifications^33,34^. However, it is still unclear whether GC content plays a role in ct-siRNA production from plant endogenous coding genes. The GC content of genome-wide coding regions follows a normal distribution, approximately ranging from 30% to 55%^35^. Interestingly, we found that ct-siRNA producing genes exhibited a higher GC content in all the mutants we tested (Supplementary Fig. 3d, e). The GC content in the flanking 1Kb regions upstream or downstream of source genes also showed positive correlations with the generation of 22-nt ct-siRNAs (Fig. 3c). In human cells, the GC content plays a central role in mRNA fate, with the translation efficiencies and stability of GC-rich mRNAs were significantly higher than AU-rich mRNAs^35^. Therefore, GC-rich mRNAs with GC ≥ 50% may become substrates for PTGS pathway and contribute to more abundant ct-siRNA production when both RNA decay and DCL4 are deficient. Conversely, regions with low GC content (GC ≤ 30%) generated lower abundances of ct-siRNAs, possibly due to limited translational efficiency (Fig. 3c). In conclusion, our results demonstrate that the selective production of ct-siRNAs is closely associated with the function, sequence length, and GC content of their source genes. This indicates that an array of gene characteristics can collectively contribute to the selective production of ct-siRNAs.

### Truncated *NIA1* and *NIA2* fragments with high GC content induce ct-siRNA production

In *Arabidopsis*, *NIA1* and *NIA2* encoding nitrate reductases, are essential for nitrate assimilation and can generate highly abundant 22-nt ct-siRNAs when nitrogen nutrition is scarce^31^. These ct-siRNAs efficiently inhibit NIA1 and NIA2 protein levels, thereby reducing energy consumption and ensuring plant survival^31^. In plants deficient in several RNA decay factors and DCL4 activity, such as *ein5-1 dcl4-2*, *fry1-6 dcl4-2*, *dxo1-1 dcl4-2*, *hen2-1 dcl4-2*, *ski2-2 dcl4-2*, and *urt1-1 dcl4-2* mutants, we observed a massive amount of 22-nt ct-siRNAs accumulated at the *NIA1* and *NIA2* loci (Fig. 2a, b, d). The question then arises: why do *NIA1* and *NIA2* genes frequently produce large quantities of ct-siRNAs to trigger endogenous gene silencing when both RNA decay and PTGS factors are deficient?

**Fig. 4:**
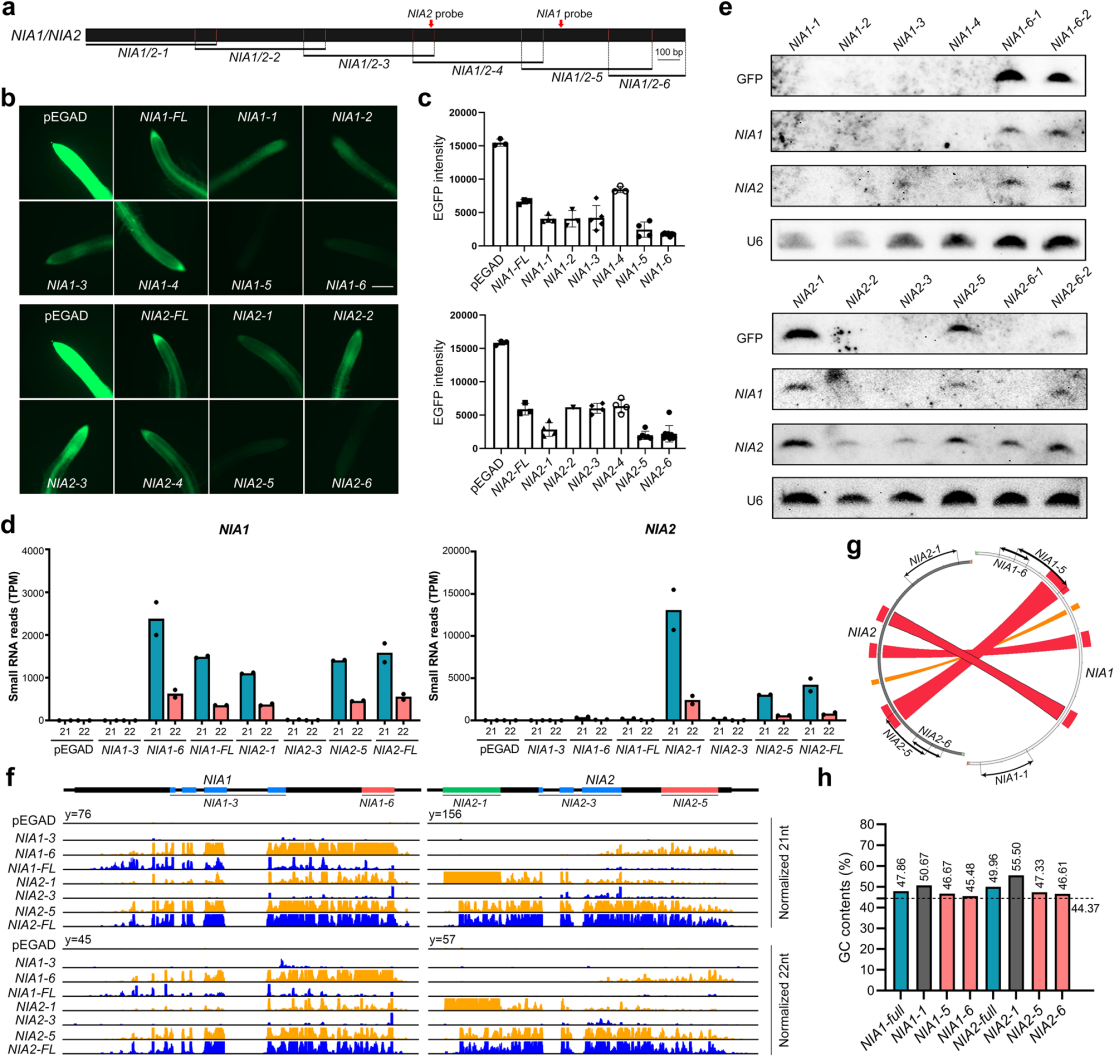
Transgenes of truncated *NIA1* and *NIA2* fragments effectively induce both gene silencing. **a** Schematic illustration of the consecutive truncated 600-nt *NIA1* and *NIA2* fragments. **b** GFP fluorescence in transgenic plants expressing truncated *NIA1* and *NIA2* fragments. Scale bar = 100 μm. **c** Fluorescence intensity was detected in transgenic plants. **d** Abundance of 21-nt and 22-nt ct-siRNAs (TPM, tags per million) accumulated at *NIA1* and *NIA2* in transgenic plants. *n* = 2 biologically independent samples. FL, full-length. **e** Northern blotting of ct-siRNAs produced from GFP, *NIA1*, and *NIA2* in transgenic plants. **f** Distribution of 21-nt and 22-nt ct-siRNAs generated from *NIA1* and *NIA2* in transgenic plants. **g** Sequence similarity between truncated *NIA1* and *NIA2* fragments. **h** GC contents of full-length and truncated *NIA1* and *NIA2* fragments.

To address this question above, we truncated the CDS sequences of *NIA1* and *NIA2* into consecutive 600-nt fragments and generated transgenic plants by expressing each fragment fused with the 35S promoter and the green fluorescent protein (GFP) sequence (Fig. 4a). Our observation revealed that only transgenic plants expressing specific fragments, like *NIA1-5*, *NIA1-6*, *NIA2-1*, *NIA2-5*, and *NIA2-6*, exhibited a strong ability to induce transgenic silencing of GFP, while other transgenic plants exhibited weak or no silencing effects (Fig. 4b). By further analyzing the sRNA-seq data from transgenic plants expressing *NIA1-3*, *NIA1-6*, *NIA2-1*, *NIA2-3*, and *NIA2-5*, we observed an obvious correlation between siRNA production from *NIA1* or *NIA2* and GFP gene silencing, but not GFP intensity in transgenic plants (Fig. 4b–d). This phenomenon may be attributed to the insufficiency of GFP fluorescence as a quantitative measure of gene silencing or to the possibility that the abundance of siRNAs precedes or lags behind the immediate state of gene silencing (Fig. 4c, d). Intriguingly, the sRNA-seq data analysis further revealed that transgenes of the aforementioned truncated *NIA1* and *NIA2* fragments could all stimulate siRNA production from both genes to varying levels. This indicates that ct-siRNAs originating from *NIA1* or *NIA2* can enforce transitive silencing of the source gene and its homologous gene (Fig. 4d). This observation was further confirmed by Northern blot analysis of two *NIA1-6* transgenic lines, which triggered siRNA production from both *NIA1* and *NIA2* (Fig. 4e). Consistent results were observed in the case of the *NIA2-1*, *NIA2-5*, and *NIA2-6* transgenic lines. However, a detailed examination of siRNA accumulation peaks at *NIA1* and *NIA2* in different transgenic lines revealed substantial differences (Fig. 4f), suggesting that multiple factors influence siRNA production.

Upon investigating sequence similarity using Circoletto with default settings (http://tools.bat.infspire.org/circoletto/), we found that *NIA2-5* is the only fragment partially homologous to *NIA1-5* (Fig. 4g), and its transgenic plant exhibited transitive siRNA production on *NIA1* (Fig. 4f). Additionally, we noticed that the GC content of full-length *NIA1* and *NIA2*, as well as all truncated fragments, exceeded the average GC content of whole transcriptome CDS sequences (44.37%) (Fig. 4h). Notably, among these fragments, *NIA2-1* with the highest GC content at 55.5% exhibited a concentrated siRNA peak within the fragment boundary (Fig. 4f, h), implying that GC content is a crucial factor influencing siRNA generation. Our genetic findings confirmed a strong correlation among GC content, siRNA production, and gene silencing. Therefore, it is crucial to calculate GC content to avoid high GC sequences and achieve efficient transgenesis, especially those sequences with a GC content exceeding 55%, which can frequently trigger gene silencing.

### ct-siRNA dynamically accumulated at different plant growth and development stages

**Fig. 5:**
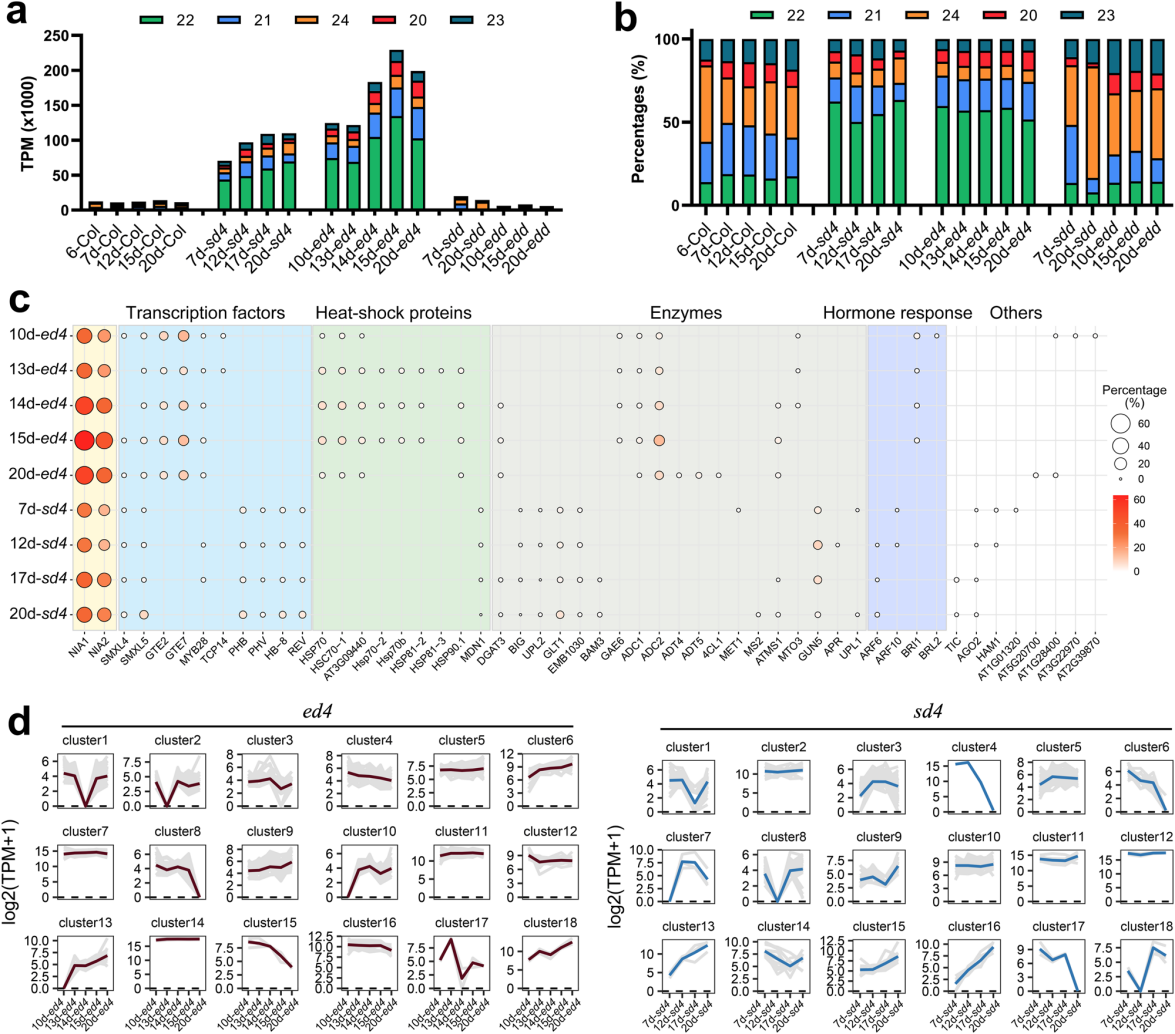
Dynamic accumulation of ct-siRNAs in *ein5 dcl4* and *ski2 dcl4* plants across different stages of growth and development. **a, b** The accumulation and percentage of 20-nt to 24-nt ct-siRNAs in *ed4* (*ein5-1 dcl4-2*) and *sd4* (*ski2-2 dcl4-2*) plants across different days. The 7th and 10th days mark the earliest time points at which homozygous mutants of *sd4* and *ed4* can be distinguished from heterozygous mutants. Both mutants die on the 21st day. **c** The percentage of top-scoring 22-nt ct-siRNA source genes ranked by accumulated 22-nt ct-siRNA abundance in each mutant. **d** Clustering analysis of genes with differentially accumulated 22-nt ct-siRNAs by their abundance in *ed4* and *sd4* plants. 583 and 423 genes with 22-nt ct-siRNA abundance TPM > 10 in at least two stages and an absolute log2FC > 1 when comparing any two stages were used.

To investigate whether the accumulation of ct-siRNAs is dynamically regulated during plant growth and development, we conducted time-series sRNA-seq on *ein5-1 dcl4-2* and *ski2-2 dcl4-2* plants. Commencing from the point at which the homozygous mutants first displayed identifiable phenotypes. We observed that 21-nt and 22-nt ct-siRNAs were rarely detected in Col-0, *ski2-2 dcl2-1 dcl4-2*, and *ein5-1 dcl2-1 dcl4-2* plants but showed a dynamic accumulation pattern when both RNA decay and DCL4 activity were deficient (Fig. 5a, b). In *ein5-1 dcl4-2* and *ski2-2 dcl4-2* plants, the expression of 22-nt ct-siRNAs gradually increased and reached its peak at 15-day-old and 20-day-old, respectively. In contrast, 21-nt ct-siRNA accumulation peaked at 14-day-old and 12-day-old, respectively (Fig. 5a, b).

We ranked the hotspot genes with high levels of ct-siRNA production to identify the source genes contributing to the dynamic accumulation of ct-siRNAs during plant growth and development (Fig. 5c). Among these genes, *NIA1* and *NIA2* consistently produced the highest proportion of 22-nt ct-siRNAs in both *ein5-1 dcl4-2* and *ski2-2 dcl4-2* plants (Fig. 5c). Interestingly, we found that 5’-3’ and 3’-5’ RNA decay factors had different effects on the production of 22-nt ct-siRNAs from various substrates (Fig. 5c). When classifying the source genes based on their functions, we found that the hotspot genes producing 22-nt ct-siRNAs in both *ein5-1 dcl4-2* and *ski2-2 dcl4-2* plants were primarily encoded transcription factors, heat-shock proteins, multiple enzymes, hormone responsive proteins, and other functional genes (Fig. 5c). The predominant production of 22-nt ct-siRNAs from genes that encoding transcription factors GTE2 and GTE7, as well as genes encoding heat-shock proteins, was only detected in *ein5-1 dcl4-2*, while genes encoding HD-ZIP transcription factors (PHB, PHV, HB-8, and REV) and several enzymes were exclusively identified in *ski2-2 dcl4-2* (Fig. 5c).

To identify distinct groups of genes that co-accumulated ct-siRNAs during different stages of plant growth and development, we performed clustering analysis of source genes based on the abundance of accumulated ct-siRNAs. Specifically, we focused on coding genes with Transcripts Per Million (TPM) levels of accumulated 22-nt ct-siRNAs greater than 10 in at least two stages, as well as that differentially accumulated with a |log2FC|> 1 when cross-comparing any two stages. Our analysis identified 18 clusters comprising 583 and 423 genes in *ein5-1 dcl4-2* and *ski2-2 dcl4-2* plants, respectively (Fig. 5d). In *ein5-1 dcl4-2* plants, we observed a continual increase in 22-nt ct-siRNA production from 145 genes (clusters 6, 9, 10, 13, and 18), which are functionally involved in regulating RNA metabolism and the cellular response to hypoxia and oxygen. Simultaneously, we found a gradual decrease in the accumulation of 22-nt ct-siRNAs from 115 genes (clusters 4, 8, and 15) that are functionally involved in photosynthesis, light harvesting, and translational elongation (Fig. 5d). In *ski2-2 dcl4-2* plants, a continuous increase expression of 22-nt ct-siRNAs was observed in 20 genes (clusters 13, 15, and 16) that played critical roles in anther, stamen, and floral development, while the abundance of 22-nt ct-siRNAs gradually decreased in 28 genes (clusters 4, 6, 14, and 17) that were not enriched in any specific biological processes (Fig. 5d). These findings suggest that the accumulation of ct-siRNAs from discrete gene loci exhibits a fluctuating pattern of changes during various stages of plant growth and development. This alternation in abundance over stages could also account for the apparent differences in ct-siRNA selective production observed in sRNA-seq snapshots.

### ct-siRNA source genes are expressed in specific cell types

In our previous study, we observed that specific endogenous coding genes, like *NIA1* and *NIA2*, accumulated large amounts of ct-siRNAs in *ein5-1 dcl4-2* and *ski2-2 dcl4-2* plants^31^. We also found that their mRNA levels remained either unchanged or upregulated in both mutants compared to Col-0 in the bulk RNA-seq^31^. However, it remains unknown whether these ct-siRNA source genes are expressed in specific cell types and whether their expression patterns contribute to the production of ct-siRNAs. In recent years, snRNA-seq has emerged as a powerful tool for studying cell-specific gene expression. Thus, we employed snRNA-seq to investigate the expression of ct-siRNA source genes at the single-cell level.

**Fig. 6:**
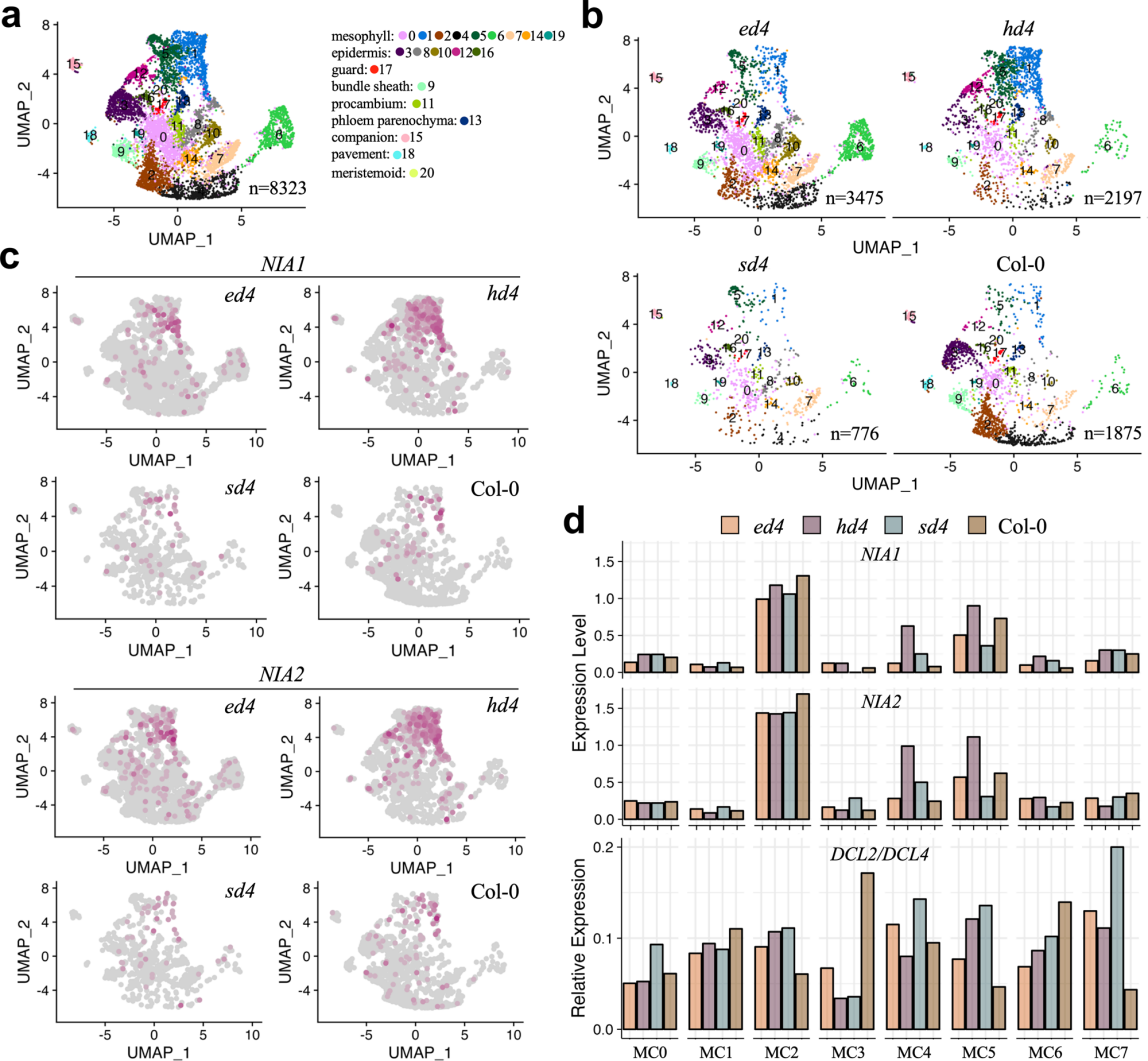
Cell-specific expression of ct-siRNA source genes. **a** UMAP visualization of seedling cell types. Each dot represents an individual cell, with color represents the respective cell type. Corresponding seedling clusters are indicated on the right. “n” indicates cell numbers. **b** UMAP visualization of Col-0, *ed4* (*ein5-1 dcl4-2*), *sd4* (*ski2-2 dcl4-2*), *and hd4* (*hen2-1 dcl4-2*) samples as shown in **a**. **c** Expression of *NIA1* and *NIA2* in each sample at the single-cell level visualized by UMAP. **d** Average expression of *NIA1* and *NIA2* and relative expression of *DCL2* versus *DCL4* in eight subtypes of mesophyll cells.

We utilized the 10X Genomics snRNA-seq platform to amplify and profile the transcriptome of cells from 21-day-old *Arabidopsis* seedlings without roots, including Col-0, *ein5-1 dcl4-2*, *ski2-2 dcl4-2*, and *hen2-1 dcl4-2* plants. After quality control at both cell and gene levels, a pool of 8323 cells with 59,950 genes were obtained from Col-0 (1875 cells), *ein5-1 dcl4-2* (3475 cells), *ski2-2 dcl4-2* (776 cells), and *hen2-1 dcl4-2* (2197 cells) plants (Fig. 6a, b). To identify distinct cell populations based on gene expression profiles, we employed graph-based clustering approach by Seurat package to identify clusters^36^. We then selected cell type-specific marker genes from the PCMDB database^37,38^ and the studies to define cell types to these clusters^39–45^. Ultimately, we manually annotated 21 clusters into 9 functional cell types (Fig. 6a, Supplementary Fig. 4). Among all cell types, mesophyll cells accounted for the largest proportion, where *NIA1* and *NIA2* showed high expression levels (Fig. 6a–c). As mesophyll cells can be further divided into subtypes such as palisade tissue and spongy tissue, we continued to define the subtypes of mesophyll cells. According to previously identified single-cell sequencing markers expressed in mesophyll^46–48^, we distinguished the mesophyll cells mainly into eight subtypes (Supplementary Fig. 5).

Analyzing the expression of ct-siRNA producing hotspot genes at the single-cell level, we observed that *NIA1* and *NIA2* were robustly expressed in the MC2 subtype of mesophyll cells (Fig. 6d, Supplementary Fig. 6), which closely resembled the palisade tissue. Notably, *dcl4-2* plants still express a *DCL4* chimeric with T-DNA sequence^31^. We also observed a notable increase in the relative expression of *DCL2* versus *DCL4* in this mesophyll subtype in *ein5-1 dcl4-2*, *ski2-2 dcl4-2*, and *hen2-1 dcl4-2* mutants compared to Col-0 plants, which might contribute to the higher abundance of 22-nt ct-siRNAs produced from *NIA1* and *NIA2* (Fig. 6d, Supplementary Figs. 6, 7). These results suggest that 22-nt ct-siRNA production may be cell type-specific. In line with this, we found that the expressions of *NIA1* and *NIA2* were downregulated in the MC2 subtype of mesophyll cells in *ein5-1 dcl4-2*, *ski2-2 dcl4-2*, and *hen2-1 dcl4-2* mutants relative to Col-0 plants (Fig. 6d). Compared to the upregulated expression levels of *NIA1* and *NIA2* observed in bulk RNA-seq (Fig. 2c), our findings suggest that gene silencing can occur at the single-cell level and may be specific to certain cell types. The expression of ct-siRNA source genes and PTGS pathway genes at single-cell level can also contribute to ct-siRNA selective generation. Thus, when the target gene fused with the 35S promoter induces gene silencing, early consideration of tissue-specific promoters should be given to achieve efficient transgenesis and molecular breeding.

## Discussion

**Fig. 7:**
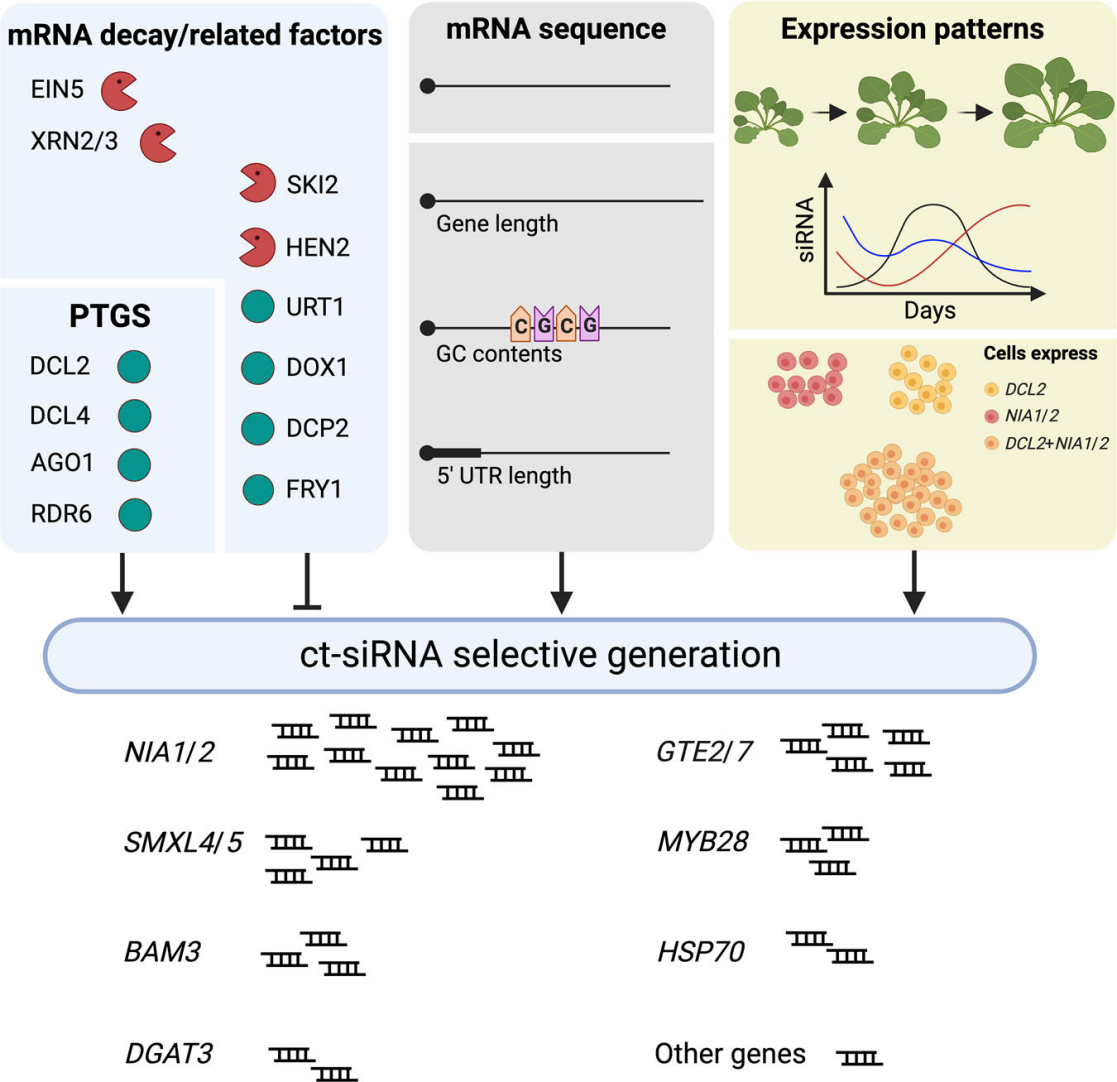
Proposed model for the selective generation of ct-siRNAs. RNA decay, PTGS, and associated factors synergistically influence ct-siRNA production, exerting either inhibitory or promotional effects. The accumulation of ct-siRNAs correlates with sequence length, GC content, and 5’ UTR length of their source genes. Concerning the expression of these genes, fluctuations induced by plant growth and development, combined with cell-level specificity, dictate ct-siRNA selective production. We hypothesize that the impact of each factor is proportional to the strength exhibited when considered individually.

Here we reported the production of 21-nt and 22-nt ct-siRNAs from endogenous mRNAs and uncovered particularly the synergistic inhibitory effects of mRNA decay and PTGS factors (Fig. 7). Among the RNA decay factors, HEN2, EIN5, DCP2, and the combination of EIN5 and SKI2 emerged as key players influencing/hindering the biogenesis of 21-nt ct-siRNAs. Meanwhile, other factors, including FRY1, SKI2, HEN2, DXO1, EIN5, and URT1, in conjunction with DCL4, specifically suppress the production of 22-nt ct-siRNAs. However, the PTGS factors DCL2 and DCL4 exhibited functional redundancy in ct-siRNA production, highlighting the complexity of the regulatory network involved in ct-siRNA biogenesis. The production of ct-siRNAs was influenced by the characteristics of their source genes, including gene length, 5’ UTR length and GC contents. Furthermore, snRNA-seq data analysis revealed that *NIA1* and *NIA2* exhibited a substantial accumulation of 22-nt ct-siRNAs in plants deficient in both EIN5/SKI2 and DCL4 and displayed an increased expression in a subtype of mesophyll cells, where a higher expression of *DCL2* relative to *DCL4* was observed.

RNA decay factors affect the selective generation of ct-siRNAs, and these effects generally reflect the disparities in decay at the 5’ and 3’ ends. This manifests in the clustering of ct-siRNA expression profiles, biological functions of source genes, and dynamic expression patterns throughout plant growth and development. This selective regulation mechanism may be influenced by the functional redundancy among the RNA decay factors. Our earlier studies found that plants deficient in RNA decay factors, in either the 5’-3’ or 3’-5’ direction, had no impact on ct-siRNA generation. However, simultaneous mutations of non-homologous RNA decay factors, EIN5 and SKI2, led to an overproduction of ct-siRNAs in the mutants, accompanied by severe growth defect phenotypes^13^. In this study, we employed EIN5, XRN2, and XRN3 in the 5’-3’ RNA decay direction and SKI2 and HEN2 in the 3’-5’ RNA decay direction. These factors, whether with sequence homology or functional redundancy, are speculated potential contributors to the selective generation of ct-siRNAs. Additionally, the selective production of ct-siRNAs may be influenced by the PTGS factors and their subcellular localization. Several previous studies have reported that DCL4 and DCL2 can form a dicing body in the nucleus. Notably, our recent research on phase separation has revealed that RDR6 and SGS3 can form liquid-liquid phase separation bodies in the cytoplasm^49^, thereby promoting endogenous gene silencing. While this aspect falls beyond the scope of this study, it merits attention in future investigations.

Our previous study has demonstrated that the nitrate reductase genes *NIA1* and *NIA2* produce large amounts of 22-nt ct-siRNAs to efficiently inhibit their protein levels, potentially promoting plant survival under stress conditions by conserving energy^31^. The efficient RNAi could be mediated by stronger transitivity or a substantial number of 22-nt siRNAs, capable of amplifying the silencing effect on their primary target or homologous gene, either in a cis or trans manner^50,51^. Previous study has indicated that a 500-nt overlap between homologous genes is sufficient to establish efficient and frequent transitive silencing, whereas homologies of 250-nt and 98-nt resulted in reduced and minimal co-suppression effect, respectively^52^. In this study, we found that 22-nt ct-siRNAs were frequently produced from *NIA1* and *NIA2* in plants particularly deficient in both RNA decay factors and DCL4 activity. This raises the question of whether ct-siRNAs induce transitive silencing between *NIA1* and *NIA2*. Gene silencing signal tends to expand towards the 3’ region of the transcript^52,53^. Our observation confirmed that the transgenic plants expressing the 3’ fragments of *NIA1* (*NIA1-5* and *NIA1-6*) and *NIA2* (*NIA2-5* and *NIA2-6*) efficiently induced the silencing of both genes, with ct-siRNAs enriched in the 3’ region and less spread to the 5’ region (Fig. 4f). It is widely accepted that at least 21-nt homology between genes can induce the co-suppression of homologous genes. Even though the CDS sequences of *NIA1* and *NIA2* share no more than successive 20-nt of identical sequence, it remains unclear how ct-siRNAs induce the transitivity silencing of homologous genes and which part of ct-siRNAs serve as efficient inducers. Previous studies have suggested that off-target silencing could be induced by approximately 70-nt fragments containing at least three mismatches within any 21-nt sequence shared between homologous genes^54^. Consequently, the transitivity and frequent silencing of *NIA1* and *NIA2* may be caused by ct-siRNA induced off-target silencing.

When RNA decay and/or PTGS factors are deficient, ct-siRNAs can produce from either aberrant or normal mRNA transcripts. Despite analyzing RNA-seq data from different mutants, we did not detect a notable downregulation in the expression of ct-siRNA source genes. This presented a contradiction to the co-suppression effect as the expression levels of ct-siRNA producing genes were unchanged or even upregulated. According to an important previous discovery^34^, one possible explanation is that the increased production of 21-nt and 22-nt ct-siRNAs corresponds to a decreased level of 24-nt siRNAs and reduced DNA methylation, leading to upregulated gene expression. On the other hand, the recent development of single-cell transcriptome sequencing technology allows us to measure gene expression at the single-cell level within samples encompassing multiple tissues and cell types. This technology has enabled us to find that hotspot genes with high-frequency accumulation of ct-siRNAs, like *NIA1* and *NIA2*, were predominantly expressed in mesophyll cells. Interestingly, we have also found that *DCL2* showed a higher expression level than other subtypes of mesophyll cells, which may provide an explanation for the selective production of 22-nt ct-siRNAs from *NIA1* and *NIA2*. More importantly, we observed the downregulated expression of *NIA1* and *NIA2* in the same subtype of mesophyll cells, aligning with our initial expectation that the production of abundant ct-siRNAs would decrease the expression of their source transcripts. Furthermore, we observed that downregulated expression of ct-siRNA source genes in specific cells might be compensated by upregulated expression of these transcripts in a variety of other cell types (Supplementary Fig. 7), resulting in their unchanged or increased expression in bulk RNA-seq data. Our results suggest that gene silencing induced by ct-siRNAs potentially occur in specific cells, and whether this triggers compensatory upregulation of genes in neighbouring cells is an interesting biological question. Given the lack of spatial information in single-cell transcriptomics, there is an urgent need for further research to leverage the maturation of spatial transcriptomics and spatial small RNA detection technologies to address this limitation.

As a fundamental surveillance mechanism, RNA decay eliminates aberrant mRNAs, preventing them from being captured by the PTGS pathway and ultimately processed into rogue ct-siRNAs. The fate of aberrant mRNAs, whether they undergo decay or are silenced by ct-siRNAs, may be determined by various factors involved in RNA decay and PTGS pathways, sequence composition, biological function, and cell-specific expression of ct-siRNA source genes. It is unclear how a single factor may affect ct-siRNA selective production, while multiple factors should be considered in both qualitatively and quantitatively.

## Methods

### Plant materials and growth conditions

The *Arabidopsis* plants of the Columbia (Col-0) accession were exclusively used. Commercially available Murashige and Skoog (MS) medium, along with nitrogen-depleted MS salt obtained from Phyto Technology Laboratories (Catalog: M524, M531), to prepare the full-nutrition MS medium and nitrogen-depleted medium (pH 5.7–5.8, 1% sucrose, 10 g/L agar), respectively. Seeds were surface-sterilized and plated on the medium^55^. Seeds pretreated with stratification for 3 days at 4 °C were kept in the greenhouse for another 6–7 days (22 °C, 16 h/8 h photoperiod) before transferring the seedlings to the soil or phenotyping.

### Genetic analysis and genotyping

The mutants and transgenic materials employed in this study were either maintained in our laboratory or purchased from SALK. The *ein5-1* alleles were derived from an x-ray mutagenized population (ecotype Col-0)^56^. The T-DNA insertional mutant *ski2-3* was acquired from SALK and subsequently validated by PCR amplification^57^. Point mutations including *rdr6-11*^58^, *ago1-47*, *ago1-45*^59^ and *hen1-8*^60^ were genotyped. The homozygous double and triple mutants (*dcl2-1* *dcl4-2*^61^, *ein5-1 dcl4-2*, *ski2-2 dcl4-2*, *ein5-1 ski2-3, ein5-1 dcl4-2 dcl2-1*, *ski2-2 dcl4-2 dcl2-1*, *ein5-1 dcl4-2 ago1-45*, *ein5-1 dcl4-2 ago1-27*^31^) were generated through genetic crosses and identified from the F_2_ or F_3_ populations. Each mutation was confirmed by PCR-based genotyping and phenotypic analysis, or through the use of antibiotic-resistant markers. To generate the *ein5-1 ski2-3 dcl4-2 dcl2-1* quadruple mutant, we genotyped the F_2_ and F_3_ plants propagated from the cross between *ein5-1 ski2-3* hemizygote and *dcl4-2 dcl2-1*. While no *ein5-1 ski2-3 dcl4-2* plant was verified from the segregating population derived from the *ein5-1 ski2-3* hemizygote and *dcl4-2 dcl2-1* cross. In these experiments, the genotyping of *ski2-3, dcl4-2*, and *dcl2-1* loci were conducted via PCR, and the *ein5-1* mutation (1-bp deletion, frameshift) was confirmed through ethylene-related phenotyping^62^ and validated through Sanger sequencing.

### RNA-seq and sRNA-seq analysis

RNA-seq and sRNA-seq data analysis was performed as described in our previous study^31^.

### Gene enrichment analysis

Gene enrichment analysis was performed using the BiNGO plugin^63^ of Cytoscape software^64^ with default parameters.

### GC content analysis

We split the coding sequences of the *Arabidopsis* reference genome (TAIR10, https://www.arabidopsis.org/) into 100-bp bins and calculated the GC contents, which were used to visualize the distribution of the whole genome GC content. In this study, GC content ≤ 30%, 30% < GC content < 50%, and GC content ≥ 50%, were defined as low, medium, and high GC regions, respectively.

### Single nucleus data preprocessing and analysis

The leaf tissue of 21-day-old Col-0, *ein5-1 dcl4-2*, *ski2-2 dcl4-2* and *hen2-1 dcl4-2* plants were harvested. We used the 10X Genomics snRNA-seq platform (http://10xgenomics.com/) to profile over 15,000 nuclei. The FASTQ files were generated from Illumina BCL files using the *mkfastq* function of Cell Ranger (version 6.1.2) (http://10xgenomics.com/) and processed to count matrix by *count* pipeline. The R package Seurat (version 3.1.5)^65^ was used to conduct single-cell data analysis. After filtering out low-quality genes in each nucleus, the retained 8757 nuclei with the percentage of mitochondrial genes (percent.mt < 5) and chloroplast genes (percent.ct < 10) were used to carry out the downstream analysis. The Seurat package was used to identify distinct cell populations based on gene expression profiles^36^. Cell populations were manually annotated to the functional cell-type clusters combining the cell markers from the PlantscRNAdb and PCMDB database^37,38^. The “FindSubCluster” function with resolution = 0.6 was used to identify subclusters in mesophyll cell publications.

### Transgenic materials

We truncated *NIA1* and *NIA2* CDS sequences to the consecutive 600-nt fragments and fused each truncated fragment with 35S promoter and green fluorescent protein (GFP) sequence to construct transgenic materials. The sequences of truncated *NIA1* and *NIA2* CDS fragments of Fig. 4a are described in Supplementary Data 1.

### Statistics and reproducibility

Genes that differentially accumulated 21-nt or 22-nt ct-siRNAs were identified by comparing the mutants deficient in RNA decay factors and/or DCL4 activity, against the Col-0 using the R package – Deseq2 (version 1.38.3)^66^, with a cutoff of *padj* < 0.05 and absolute log2FC (Fold Change) > 1. At least three biological replicates were used for these analyses.

Statistical analyses were conducted on source gene sequence length, 5’ UTR length, 3’ UTR length, intron number, and GC ratio using R (version 4.2.2). The source data for gene sequence length and 5’ UTR length are provided in Supplementary Data 1. Statistical significance was determined through a two-tailed Student’s *t*-test (***p < 0.001, ****p < 0.0001) by comparing genes enriched in 21-nt or 22-nt ct-siRNAs with non-22-nt siRNA-producing genes.

Statistical analysis of fluorescence intensity in transgenic plant expressing each truncated *NIA1* and *NIA2* fragment was performed using GraphPad Prism 9. At least three technical replicates were detected for each sample, except for the transgenic plant expressing *NIA2-2*, which had one replicate. Error bars were represented using the standard deviation (SD). Additionally, the abundance of sRNAs produced from transgenic plant expressing each truncated *NIA1* and *NIA2* fragment was detected with two biological replicates.

Genes were chosen for clustering analysis based on the accumulation of 22-nt ct-siRNAs, with TPM > 10 in at least two stages, and an absolute log2FC > 1 when comparing any two stages. Each stage for the *ein5-1 dcl4-2* and *ski2-2 dcl4-2* mutant plants had one replicate. A total of 18 clusters were identified among the stages of the *ein5-1 dcl4-2* and *ski2-2 dcl4-2* plants using R package – pheatmap (version 1.0.12), respectively.

### Reporting summary

Further information on research design is available in the Nature Portfolio Reporting Summary linked to this article.

### Supplementary information

Supplementary Figs.

Description of Supplementary Materials

Supplementary Data 1

Reporting Summary

## Data Availability

The part of raw sRNA-seq data and all RNA-seq data used in this study have been published by our previous work^31^, which were deposited on the NCBI Gene Expression Omnibus^67^ under the accession GSE136164. The raw sRNA-seq and snRNA-seq data generated by this study can be accessed on the National Genomics Data Center under the BioProject PRJCA024518. The numerical source values underlying Fig. 1a, b, Fig. 2a–c, e, f, Fig. 3a–c, Fig. 4a, c, d, h, Fig. 5a–d, and Fig. 6a, d can be found in Supplementary Data 1. All other data related to this study can also be available upon reasonable request to the corresponding or 1^st^ author. Uncropped and unedited gel images are added in Supplementary Fig. 8.
