# Author Correction: Multiple factors and features dictate the selective production of ct-siRNA in *Arabidopsis*

**DOI:** 10.1038/s42003-024-06241-2

**Published:** 2024-05-06

**Authors:** Li Feng, Wei Yan, Xianli Tang, Huihui Wu, Yajie Pan, Dongdong Lu, Qianyan Ling-hu, Yuelin Liu, Yongqi Liu, Xiehai Song, Muhammad Ali, Liang Fang, Hongwei Guo, Bosheng Li

**Affiliations:** 1 Peking University Institute of Advanced Agricultural Sciences, Shandong Laboratory of Advanced Agriculture Sciences in Weifang, Weifang, Shandong 261325 China; 2 Institute of Plant and Food Science, Department of Biology, Southern University of Science and Technology, Shenzhen, Guangdong 518055 China

**Keywords:** RNA decay, siRNAs

Correction to: *Communications Biology* 10.1038/s42003-024-06142-4, published online 18 April 2024

The original version of the article, the order of the given name and family name of the first author, Li Feng, is inverted, appearing as ‘Feng Li’ in the online version. The correct name for the first author should be “Li, Feng”, instead. This has now been corrected in the PDF and HTML versions of the Article.

