# Supplementary material for: Multiple factors and features dictate the selective production of ct-siRNA in Arabidopsis
Source: Commun Biol. 2024 Apr 18;7:474. doi: 10.1038/s42003-024-06142-4 (PMC11026412; doi:10.1038/s42003-024-06142-4)
Supplement: Supplementary file 1 — Supplementary Figs. [file 42003_2024_6142_MOESM1_ESM.pdf]

Supplementary Figures

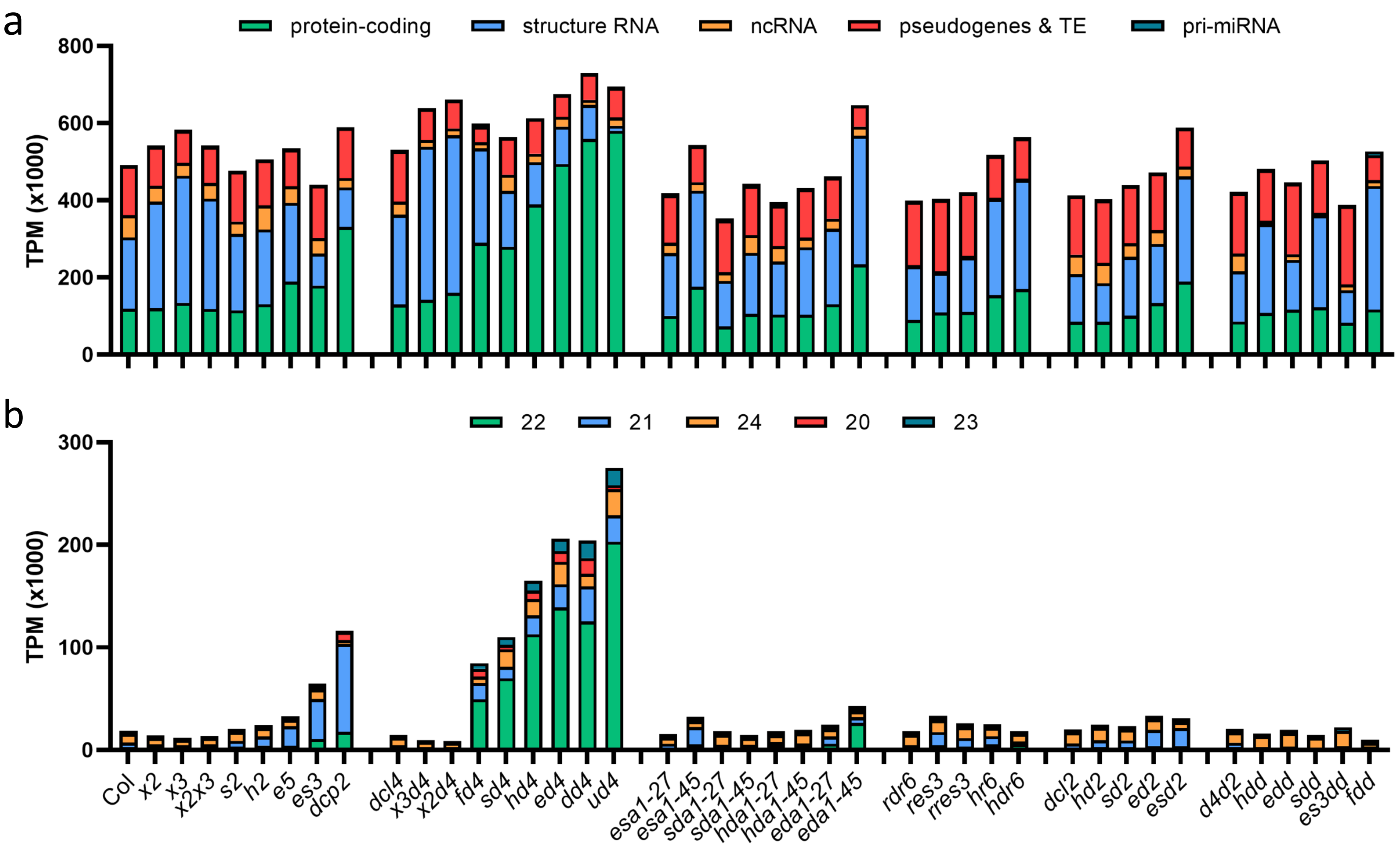

Supplementary Fig. 1 a. The expression (Transcript per million, TPM) levels of siRNAs derived from various siRNA-generating loci, including protein-coding genes, structure RNAs (ribosomal RNAs and transfer RNAs based on Rfam database), non-coding RNAs, pseudogenes & TEs, and pri-miRNAs. Sample abbreviation is the same as Fig. 1. **b.** The expression levels of ct-siRNAs with lengths ranging from 20-nt to 24-nt. Only reads produced from the antisense strand of protein-coding genes, representing ct-siRNAs, were calculated. The expression is displayed in TPM.

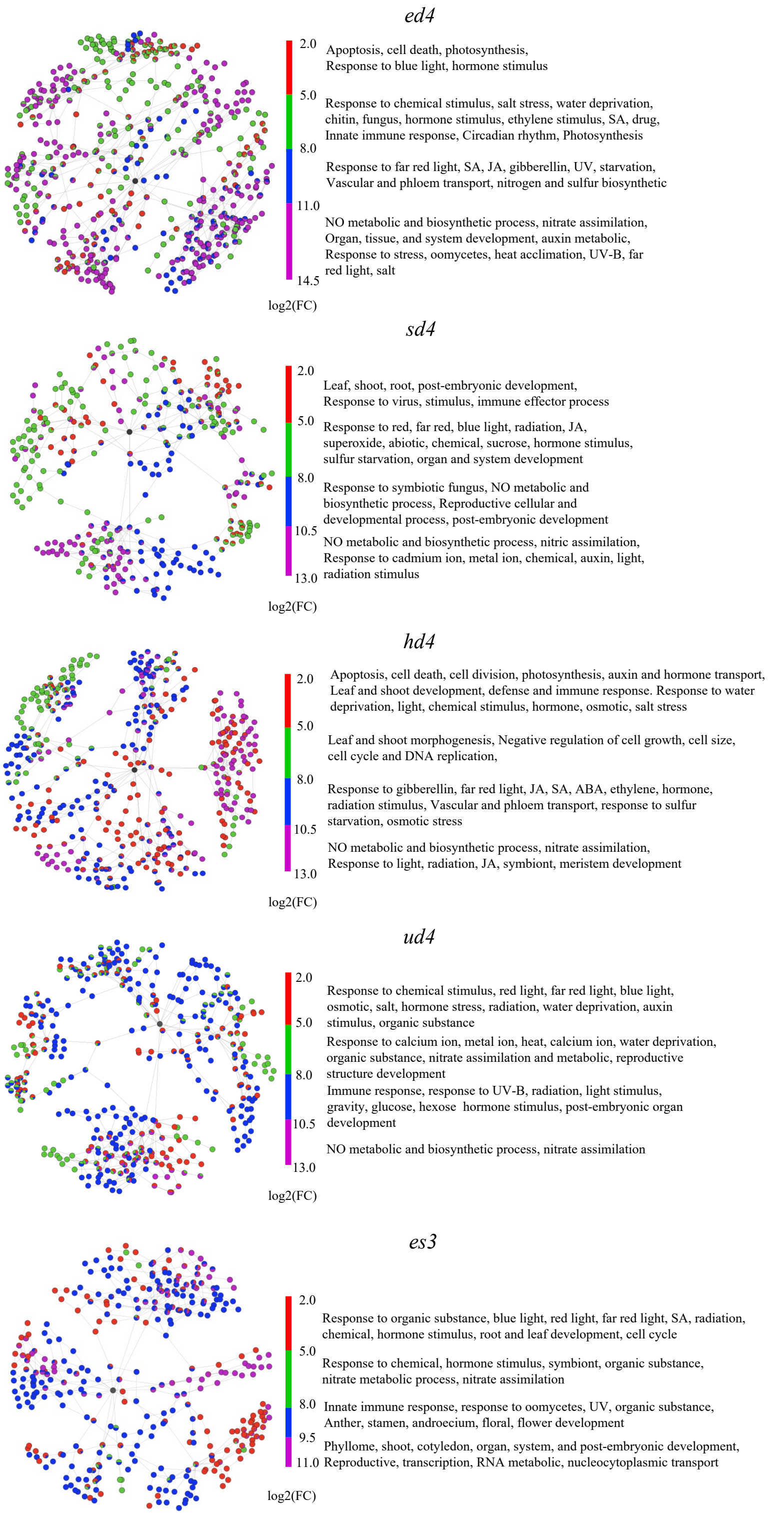

Supplementary Fig. 2 An increased fold-change range of ct-siRNA accumulation in plants deficient in RNA decay factors and/or DCL4 activity was related to the biological functions of their source gene. Red, green, blue, and purple color bars represent different ranges of increased fold (log2) of ct-siRNA expression. Left figure depicts the Gene Ontology (GO) annotation network of ct-siRNA source genes. Points with multiple colors indicate functionally overlapped genes. Right part shows the biological processes involved by ct-siRNA source genes.

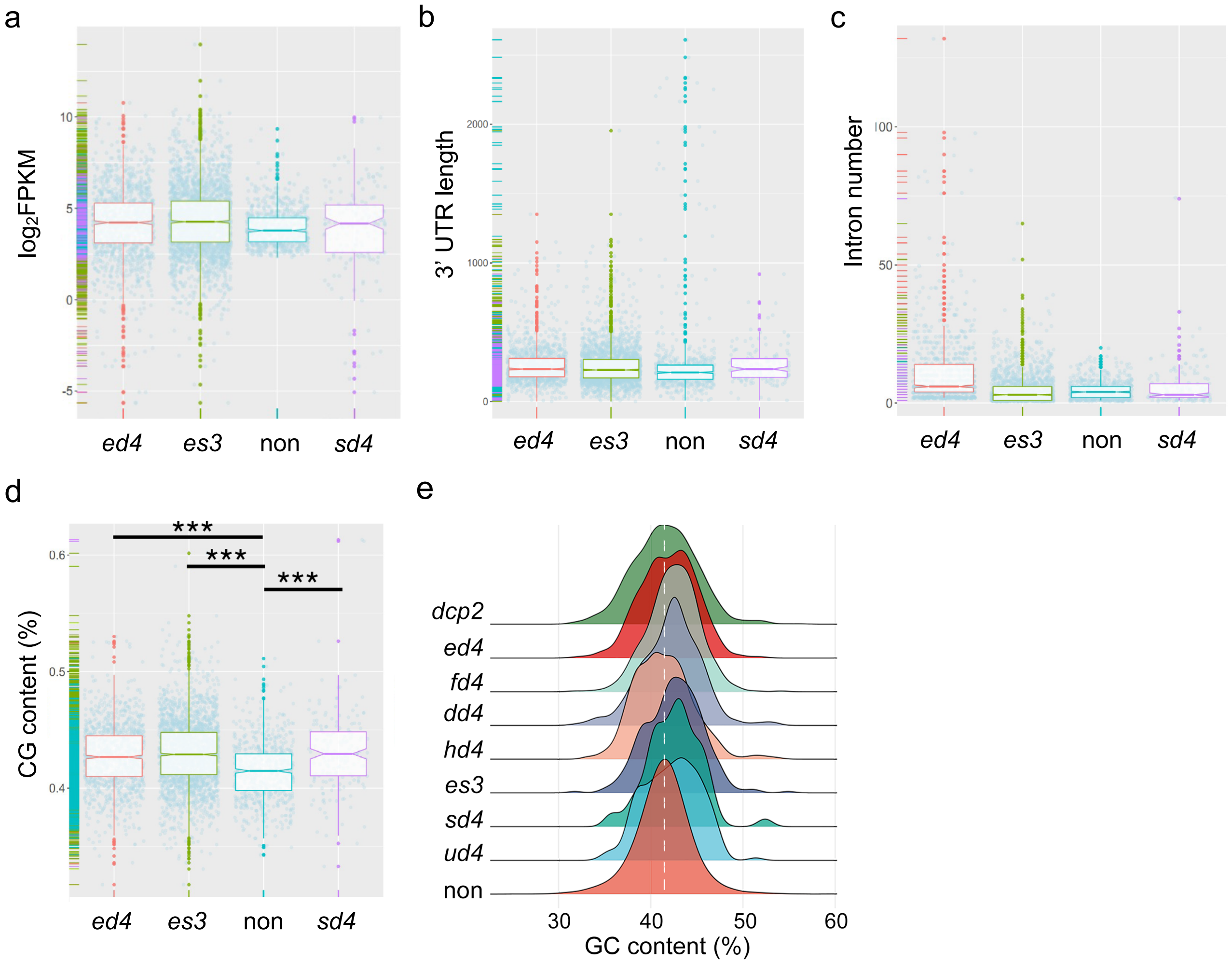

Supplementary Fig. 3 The production of ct-siRNAs was not correlated with 3' UTR length, intron number, and expression levels of their source genes. Comparison of the characteristics of 21-nt and 22-nt ct-siRNA source genes in *ed4* (*ein5-1 dcl4-2*), *sd4* (*ski2-2* dcl4-2), *es3* (*ein5-1 ski2-3*), and non-22-nt ct-siRNA-generating genes. **a.** Expression levels. **b.** 3' UTR length. **c.** intron number. **d.** GC content. Statistical significance was calculated by two-tailed Student's t-test (***, $p < 0.001$). **e.** Distribution of GC content within coding sequences of ct-siRNA source genes. Sample abbreviation is the same as Fig. 1.

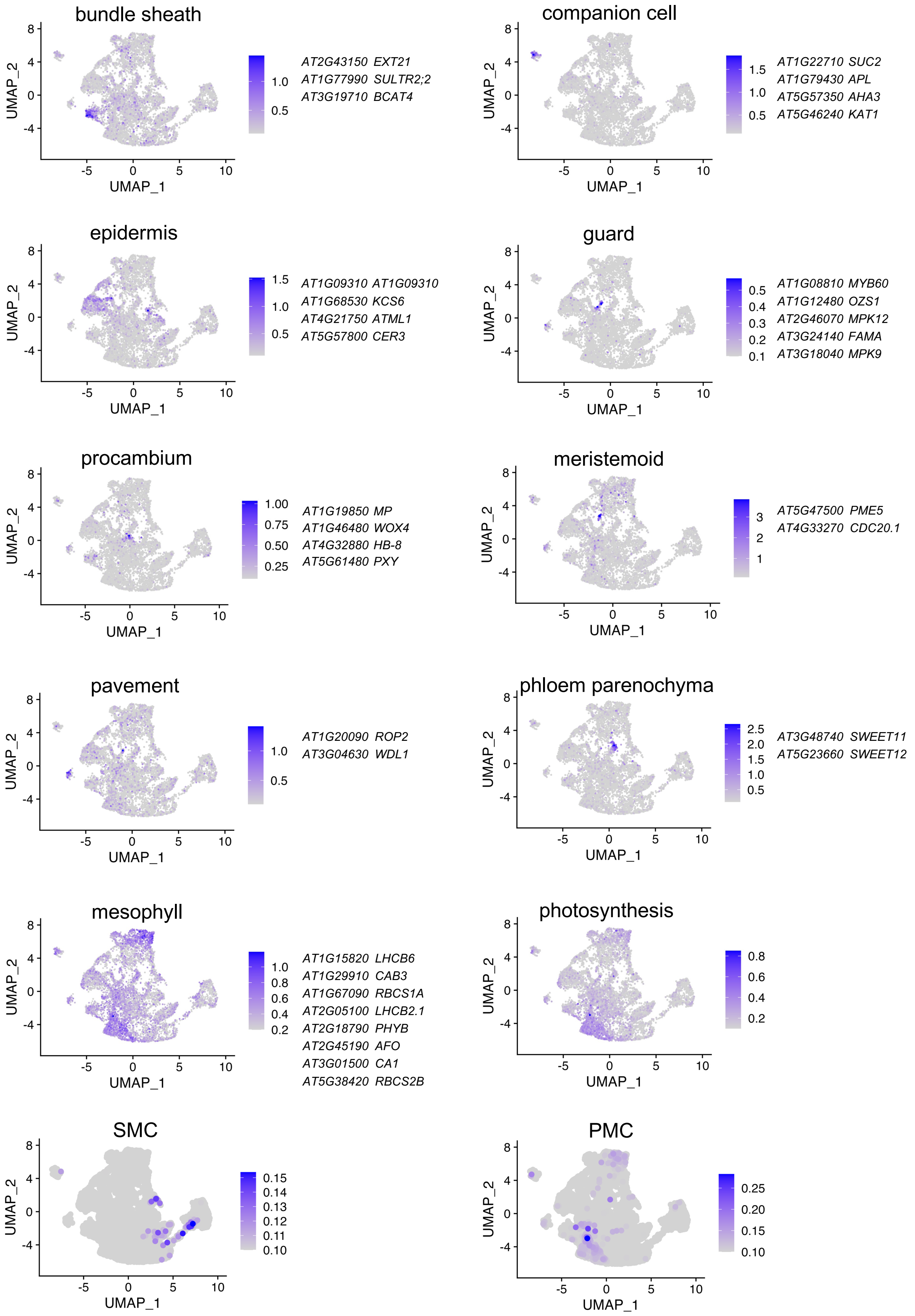

Supplementary Fig. 4 Cell type-specific marker genes was used to define cell types for identified cell populations. The collective expression of marker genes for each cell type was visualized by UMAP. Photosynthesis, SMC (spongy mesophyll cells), and PMC (palisade mesophyll cells) marker genes were listed in Supplementary Data 1.

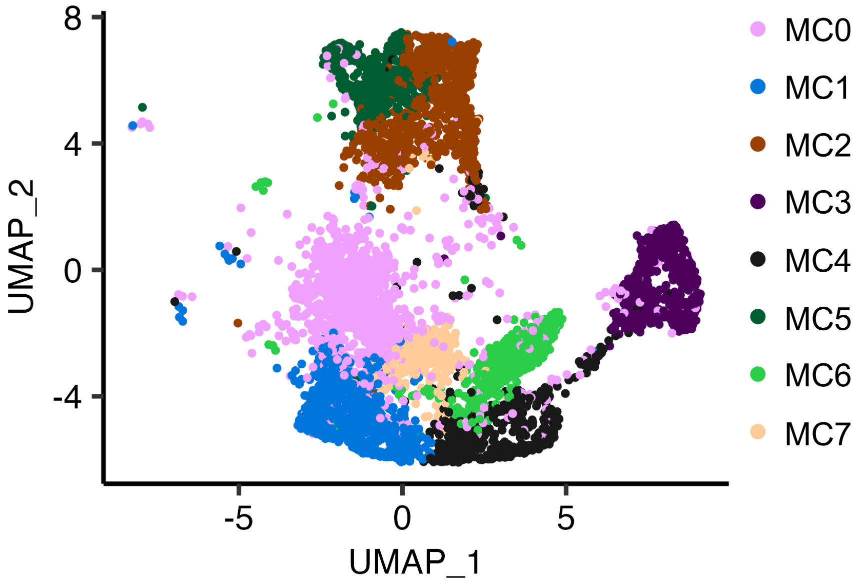

Supplementary Fig. 5 snRNA-seq defines eight subtypes of mesophyll cells.

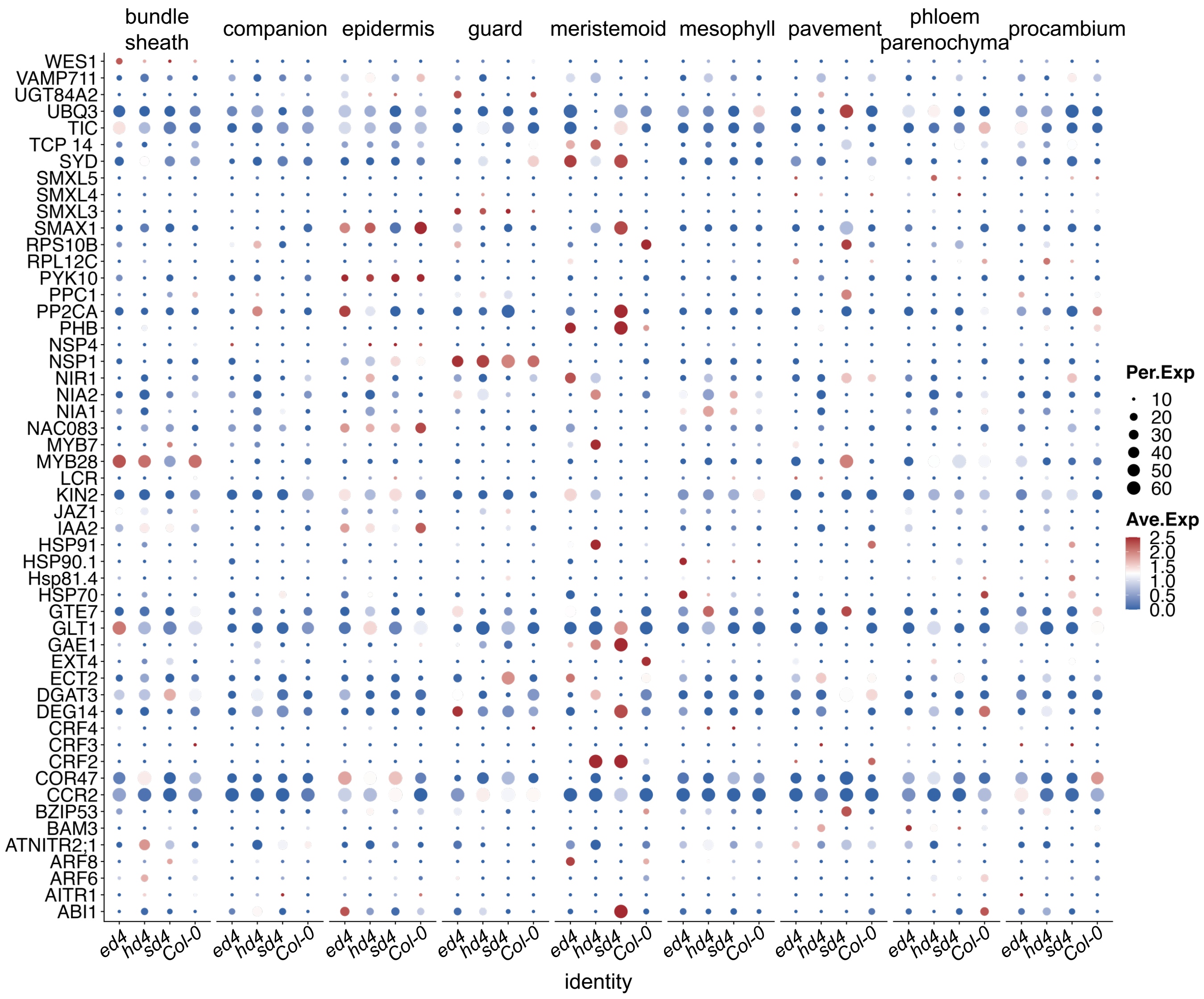

Supplementary Fig. 6 Average cell-type expression of 52 hotspot genes in three mutants and the Col-0 plant.

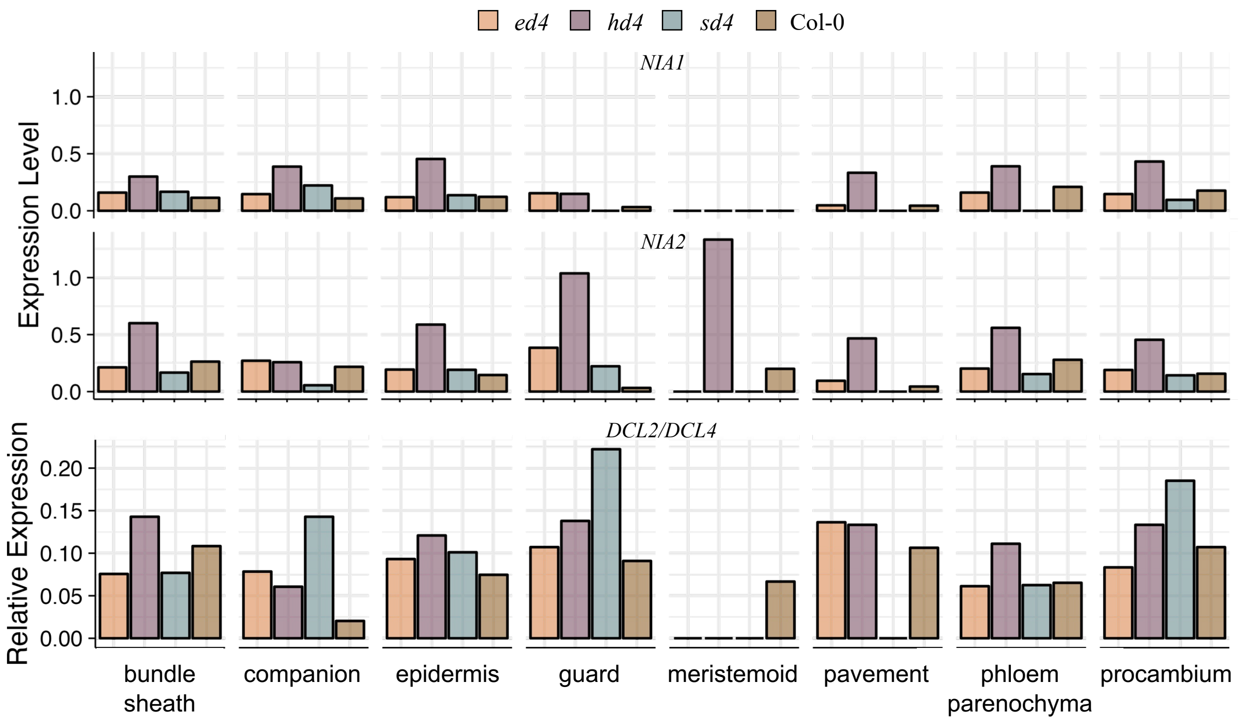

Supplementary Fig. 7 Average cell-type expression of *NIA1* and *NIA2* and relative expression of *DCL2* versus *DCL4* in three mutants and the Col-0 plant.

Supplementary Fig. 8 Uncropped and unedited gel images, refer to Fig. 4e.

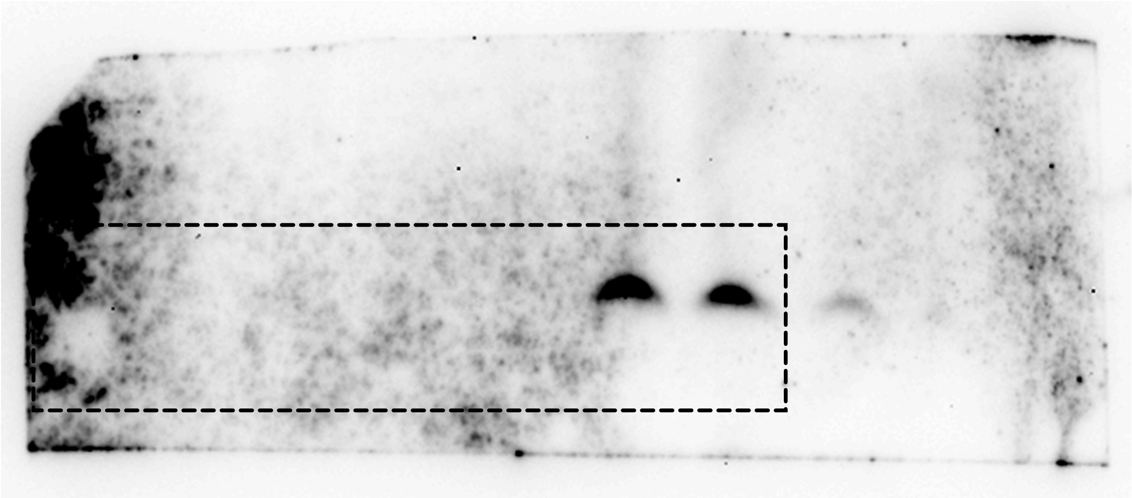

NIA1-1, NIA1-2, NIA1-3, NIA1-4, NIA1-6-1,NIA1-6-2. Probe: GFP

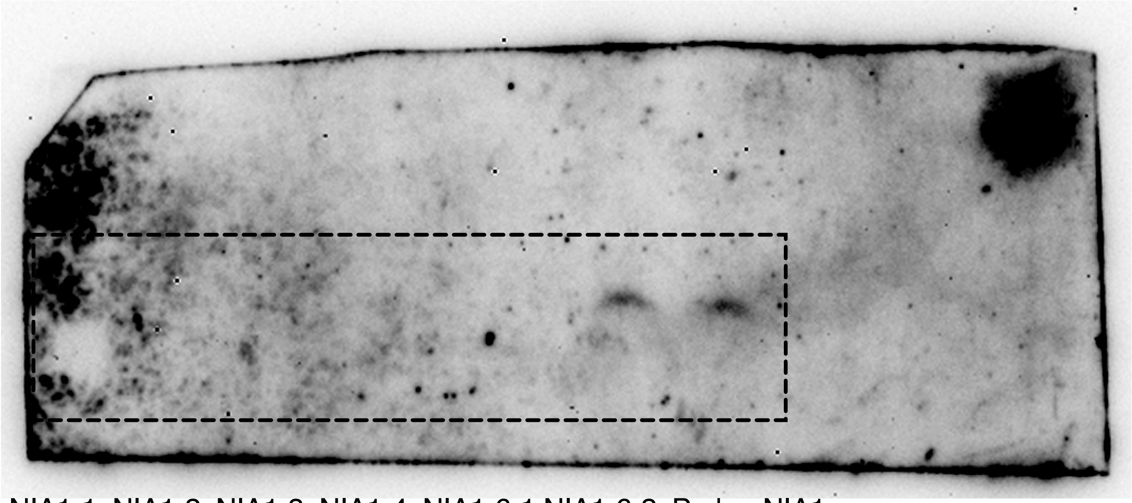

NIA1-1, NIA1-2, NIA1-3, NIA1-4, NIA1-6-1,NIA1-6-2. Probe: NIA1

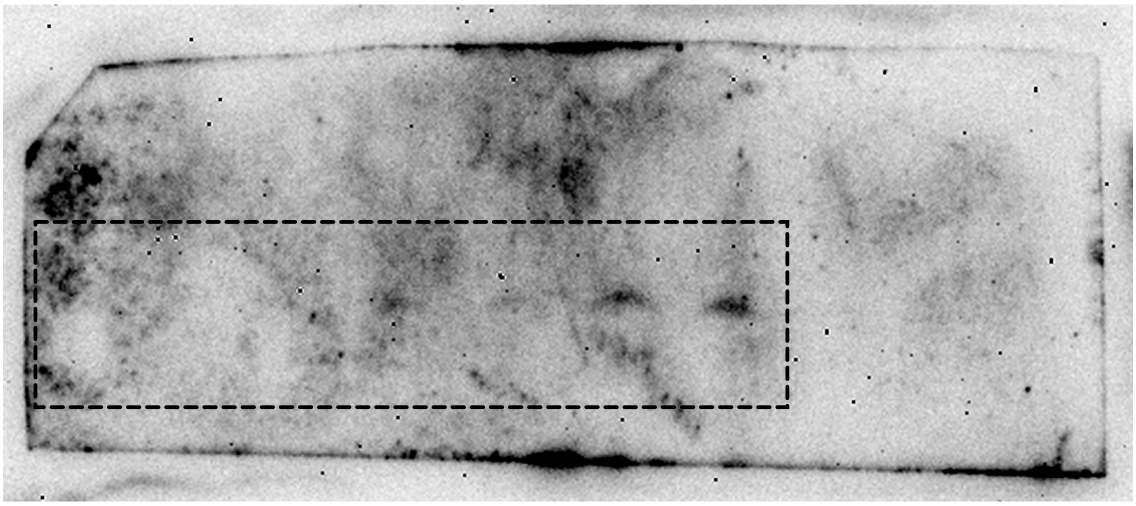

NIA1-1, NIA1-2, NIA1-3, NIA1-4, NIA1-6-1,NIA1-6-2. Probe: NIA2

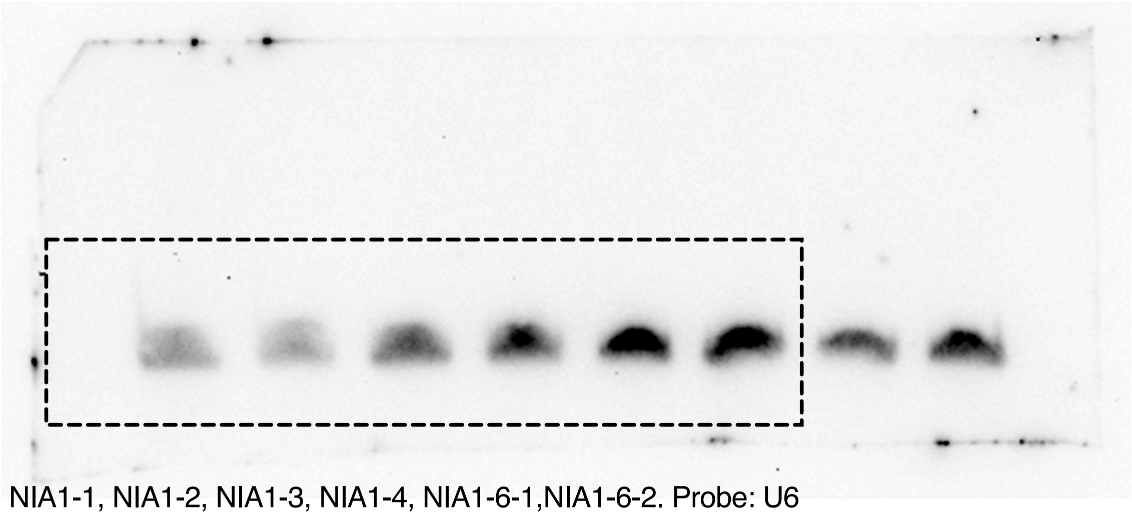

NIA1-1, NIA1-2, NIA1-3, NIA1-4, NIA1-6-1,NIA1-6-2. Probe: U6

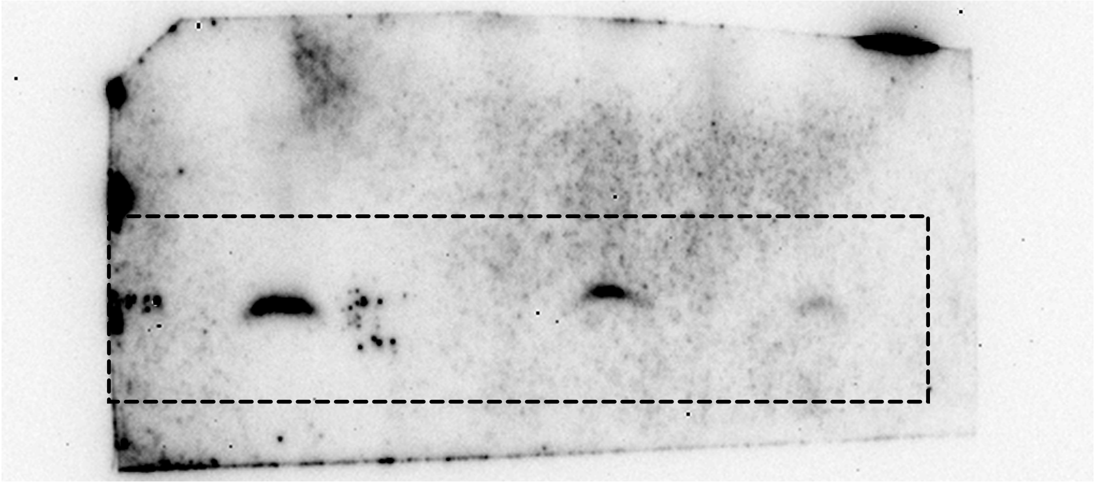

NIA2-1, NIA2-2, NIA2-3, NIA2-5, NIA2-6-1,NIA2-6-2. Probe: GFP

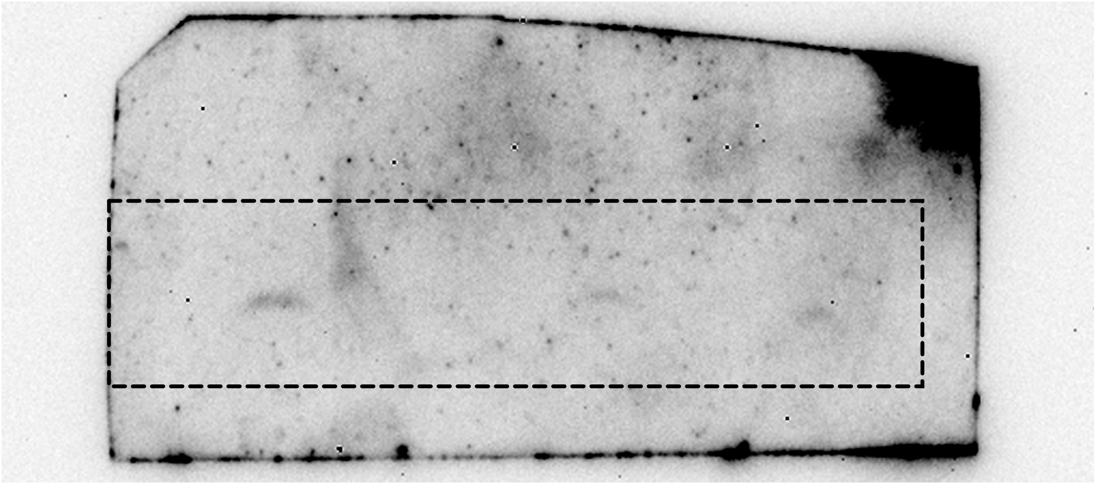

NIA2-1, NIA2-2, NIA2-3, NIA2-5, NIA2-6-1,NIA2-6-2. Probe: NIA1

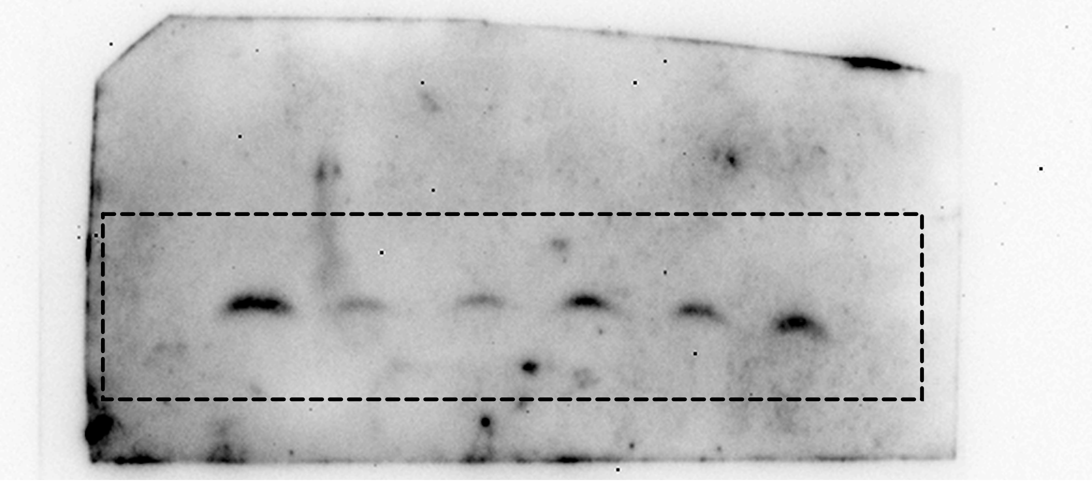

NIA2-1, NIA2-2, NIA2-3, NIA2-5, NIA2-6-1,NIA2-6-2. Probe: NIA2

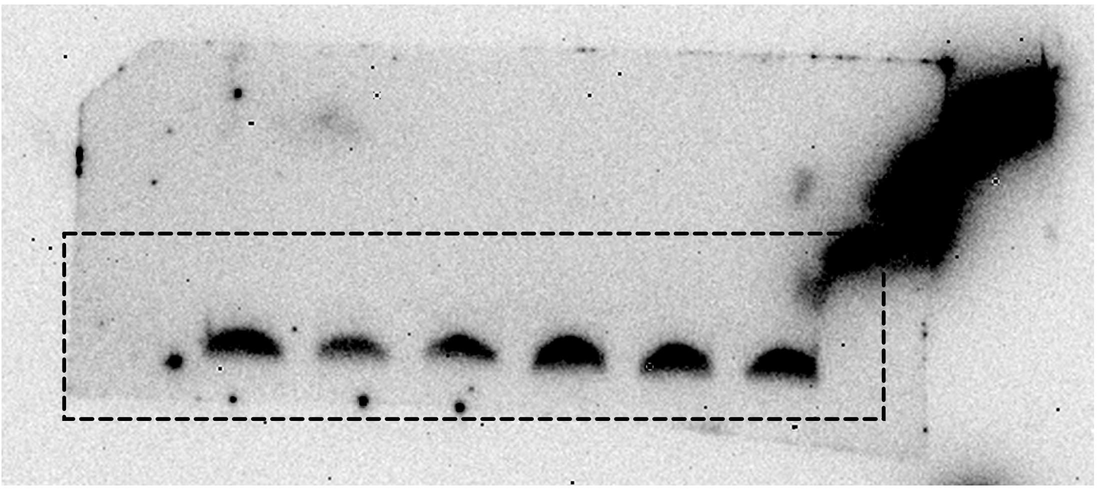

NIA2-1, NIA2-2, NIA2-3, NIA2-5, NIA2-6-1,NIA2-6-2. Probe: U6
