# Supplementary material for: Multiple factors and features dictate the selective production of ct-siRNA in Arabidopsis
Source: Commun Biol. 2024 Apr 18;7:474. doi: 10.1038/s42003-024-06142-4 (PMC11026412; doi:10.1038/s42003-024-06142-4)
Supplement: Supplementary file 2 — Description of Supplementary Materials [file 42003_2024_6142_MOESM2_ESM.docx]

**Description of Additional Supplementary Files**

**File name:** Supplementary Data 1

**Description:** The numerical source values underlying Fig. 1a, b, Fig. 2a-c, e, f, Fig. 3a-c, Fig. 4a, c, d, h, Fig. 5a-d, and Fig. 6a, d
