# Supplementary material for: Multiple factors and features dictate the selective production of ct-siRNA in Arabidopsis
Source: Commun Biol. 2024 Apr 18;7:474. doi: 10.1038/s42003-024-06142-4 (PMC11026412; doi:10.1038/s42003-024-06142-4)
Supplement: Supplementary file 4 — Reporting Summary [file 42003_2024_6142_MOESM4_ESM.pdf]

nature portfolio

Corresponding author(s): Bosheng Li
COMMSBIO-23-4312-T

Last updated by author(s): 2024/01/18

Reporting Summary

Nature Portfolio wishes to improve the reproducibility of the work that we publish. This form provides structure for consistency and transparency in reporting. For further information on Nature Portfolio policies, see our Editorial Policies and the Editorial Policy Checklist.

Statistics

For all statistical analyses, confirm that the following items are present in the figure legend, table legend, main text, or Methods section.

n/a	Confirmed	
☐	☒	The exact sample size (*n*) for each experimental group/condition, given as a discrete number and unit of measurement
☐	☒	A statement on whether measurements were taken from distinct samples or whether the same sample was measured repeatedly
☐	☒	The statistical test(s) used AND whether they are one- or two-sided<br>*Only common tests should be described solely by name; describe more complex techniques in the Methods section.*
☒	☐	A description of all covariates tested
☒	☐	A description of any assumptions or corrections, such as tests of normality and adjustment for multiple comparisons
☐	☒	A full description of the statistical parameters including central tendency (e.g. means) or other basic estimates (e.g. regression coefficient) AND variation (e.g. standard deviation) or associated estimates of uncertainty (e.g. confidence intervals)
☐	☒	For null hypothesis testing, the test statistic (e.g. *F*, *t*, *r*) with confidence intervals, effect sizes, degrees of freedom and *P* value noted<br>*Give P values as exact values whenever suitable.*
☒	☐	For Bayesian analysis, information on the choice of priors and Markov chain Monte Carlo settings
☒	☐	For hierarchical and complex designs, identification of the appropriate level for tests and full reporting of outcomes
☒	☐	Estimates of effect sizes (e.g. Cohen's *d*, Pearson's *r*), indicating how they were calculated

Our web collection on statistics for biologists contains articles on many of the points above.

Software and code

Policy information about availability of computer code

Data collection	No software was used for data collection
Data analysis	We have provided a comprehensive description of the availability of the softwares and online tools in the manuscript as follows:<br>RNA-seq and sRNA-seq data analysis: Previous study (Wu, H. et al. Plant 22-nt siRNAs mediate translational repression and stress adaptation. Nature 581, 89–93 (2020). For mRNA-seq: Raw reads were filtered by Trimmomatic (v0.38) with default parameter. Then filtered reads were aligned to the Arabidopsis Col-0 genome (TAIR10 release) using tophat2 (v2.1.1) program with 2 mismatches allowed. The differential expressed genes were called by cuffdiff (v2.2.1).<br>For sRNA-seq: Raw reads were filtered by fastx_toolkit (v0.0.14) . The 18-26 nt clean reads were mapped to structural RNA database .<br>The R package–Deseq2 (https://bioconductor.org/packages/release/bioc/html/DESeq2.html) was utilized to identify genes. Gene enrichment analysis was performed by using: 1. BiNGO plugin (https://www.psb.ugent.be/cbd/papers/BiNGO/Home.html) 2. Cytoscape software (https://cytoscape.org/).<br>GC content analysis was done by using TAIR10, (https://www.arabidopsis.org/).<br>For Single nucleus data preprocessing and analysis we used 10X Genomics snRNA-seq platform to profile nuclei (http://10xgenomics.com/); FASTQ files were generated from Illumina BCL files using the mkfastq function of Cell Ranger (version 6.1.2) (http://10xgenomics.com/); and R package Seurat (version 3.1.5) used to conduct single cell data analysis (https://satijalab.org/seurat).<br>PlantscRNAdb (http://ibi.zju.edu.cn/plantscrnadb/) and PCMDB database (https://www.tobaccodb.org/pcmdb/homePage) were used to annotate the functional cell-type clusters.

For manuscripts utilizing custom algorithms or software that are central to the research but not yet described in published literature, software must be made available to editors and reviewers. We strongly encourage code deposition in a community repository (e.g. GitHub). See the Nature Portfolio guidelines for submitting code & software for further information.

Data

Policy information about availability of data

All manuscripts must include a data availability statement. This statement should provide the following information, where applicable:

- Accession codes, unique identifiers, or web links for publicly available datasets
- A description of any restrictions on data availability
- For clinical datasets or third party data, please ensure that the statement adheres to our policy

RNA-seq data used in this study have been published by our previous work, accessed on the NCBI Gene Expression Omnibus under the accession GSE136164. sRNA-seq data and snRNA-seq data can be accessed on the National Genomics Data Center under the BioProject PRJCA018887.

Research involving human participants, their data, or biological material

Policy information about studies with human participants or human data. See also policy information about sex, gender (identity/presentation), and sexual orientation and race, ethnicity and racism.

Reporting on sex and gender	Not applicable
Reporting on race, ethnicity, or other socially relevant groupings	Not applicable
Population characteristics	Not applicable
Recruitment	Not applicable
Ethics oversight	Not applicable

Note that full information on the approval of the study protocol must also be provided in the manuscript.

Field-specific reporting

Please select the one below that is the best fit for your research. If you are not sure, read the appropriate sections before making your selection.

☒ Life sciences ☐ Behavioural & social sciences ☐ Ecological, evolutionary & environmental sciences

For a reference copy of the document with all sections, see nature.com/documents/nr-reporting-summary-flat.pdf

Life sciences study design

All studies must disclose on these points even when the disclosure is negative.

Sample size	No statistical methods were used to predetermine sample sizes. The required experimental sample sizes were estimated based on previously established protocols in the field. For all small RNA-seq, RNA-seq, qRT-PCR, and Single nucleus sequencing, 3 replicates collecting each from 15 individual seedlings of each genotype were used. The sample sizes were deemed adequate as the experimental results were consistency and reproducible.
Data exclusions	No data were excluded from the analysis
Replication	All of the experimental findings including RNA-seq, small RNA-seq, qRT-PCR, and Single nucleus analysis in our manuscript are reproducible
Randomization	Samples of the same mutants were randomly collected and pooled for all type of analysis.
Blinding	Blinding was not feasible in this study since the author who conducted the experiments also analyzed the data.

Reporting for specific materials, systems and methods

We require information from authors about some types of materials, experimental systems and methods used in many studies. Here, indicate whether each material, system or method listed is relevant to your study. If you are not sure if a list item applies to your research, read the appropriate section before selecting a response.

Materials & experimental systems

n/a	Involved in the study
☒	☐ Antibodies
☒	☐ Eukaryotic cell lines
☒	☐ Palaeontology and archaeology
☒	☐ Animals and other organisms
☒	☐ Clinical data
☒	☐ Dual use research of concern
☐	☒ Plants

Methods

n/a	Involved in the study
☒	☐ ChIP-seq
☒	☐ Flow cytometry
☒	☐ MRI-based neuroimaging

Dual use research of concern

Policy information about dual use research of concern

Hazards

Could the accidental, deliberate or reckless misuse of agents or technologies generated in the work, or the application of information presented in the manuscript, pose a threat to:

No	Yes
☒	☐ Public health
☒	☐ National security
☒	☐ Crops and/or livestock
☒	☐ Ecosystems
☒	☐ Any other significant area

Experiments of concern

Does the work involve any of these experiments of concern:

No	Yes
☒	☐ Demonstrate how to render a vaccine ineffective
☒	☐ Confer resistance to therapeutically useful antibiotics or antiviral agents
☒	☐ Enhance the virulence of a pathogen or render a nonpathogen virulent
☒	☐ Increase transmissibility of a pathogen
☒	☐ Alter the host range of a pathogen
☒	☐ Enable evasion of diagnostic/detection modalities
☒	☐ Enable the weaponization of a biological agent or toxin
☒	☐ Any other potentially harmful combination of experiments and agents

Plants

Seed stocks	Most of the mutants and transgenic materials used in this study were purchased from SALK.
Novel plant genotypes	To generate the ein5-1 ski2-3 dcl4-2 dcl2-1 quadruple mutant, we genotyped the F2 and F3 plants propagated from the cross between ein5-1 ski2-3 hemizygote and dcl4-2 dcl2-1. While, no ein5-1 ski2-3 dcl4-2 plant was verified from the segregating population derived from the ein5-1 ski2-3 hemizygote and dcl4-2 dcl2-1 cross. The other mutants and transgenic materials used in this study were mentioned in the material and methods section with their proper references.
Authentication	In these experiments, the genotyping of ski2-3, dcl4-2, dcl2-1 loci were conducted via PCR, and the ein5-1 mutation (1-bp deletion, frame shift) was confirmed through ethylene-related phenotyping and validated through Sanger sequencing.
